# A simple Schiff base compound as mitigator for the destruction of C-steel in HCl aqueous solutions: practical and theoretical studies

**DOI:** 10.1038/s41598-026-53317-6

**Published:** 2026-06-20

**Authors:** Mahmoud G.A. Saleh, H. Hawsawi, Salih S. Al-Juaid, K. A. Soliman, M. A. Hegazy, M. Abdallah, Syed Khalid Mustafa, Salah Abd El Wanees

**Affiliations:** 1 Chemistry Department, College of Science, Northern Border University, Arar, Saudi Arabia; 2 University College of Alwajh, University of Tabuk, Alwajh, Tabuk, Saudi Arabia; 3 Chemistry Department, Faculty of Science, King Abdulaziz University, Jeddah, 21589 Saudi Arabia; 4 Chemistry Department, Faculty of Science, Benha University, Benha, 13518 Egypt; 5 Egyptian Petroleum Research Institute, Cairo, Egypt; 6 Chemistry Department, Faculty of Science, University of Tabuk, Tabuk, Saudi Arabia; 7 Chemistry Department, Faculty of Science, Zagazig University, Zagazig, Egypt

**Keywords:** Corrosion inhibitor, Carbon steel, Hydrogen production, Impedance, Gravimetry, Gasometry, Potentiodynamic, Reverse mining, Chemistry, Materials science

## Abstract

The corrosion inhibition performance of *N-(1-methylpyrrolidin-2-ylidene) benzo[d]thiazol-2-amine* (MPBA) for carbon steel in 1.0 M HCl solution was systematically investigated using chemical and electrochemical techniques. The corrosion rate decreased markedly with increasing MPBA concentration. Inhibition efficiency increased progressively with inhibitor concentration, reaching values exceeding 97.0% at 5 mM and 298 K. The inhibition mechanism was primarily governed by the adsorption of MPBA molecules onto the carbon steel surface and was well described by the Langmuir adsorption isotherm. The negative values of the standard Gibbs free energy of adsorption (Δ*G*_ads_) confirm the spontaneous nature of the adsorption process. Depending on temperature, Δ*G*°_ads_ values ranged from − 37.75 to − 36.20 kJ mol⁻¹, indicating a mixed physisorption–chemisorption mechanism. The observed decrease in the adsorption equilibrium constant (*K*_ads_) with increasing temperature can be attributed to the partial desorption of MPBA molecules from the carbon steel surface. Density functional theory (DFT) calculations combined with the conductor-like polarizable continuum model (CPCM), along with Monte Carlo and molecular dynamics simulations, further confirmed the strong adsorption affinity of MPBA toward the steel surface, thereby supporting its excellent corrosion inhibition performance.

**Supplementary Information:**

The online version contains supplementary material available at 10.1038/s41598-026-53317-6.

## Introduction

Carbon steel is widely used in industrial applications, including oil and gas operations and mining processes, due to its favorable mechanical properties and low cost^1,2^. In mining and mineral processing, acidic solutions such as hydrochloric acid are commonly employed for ore treatment, descaling, and equipment cleaning. However, exposure to such aggressive media causes severe corrosion of steel surfaces, leading to material degradation and significant economic losses^3^. To mitigate these effects, corrosion inhibitors are widely applied, with increasing emphasis on environmentally friendly and cost-effective organic compounds containing heteroatoms that enhance adsorption on metal surfaces^4–12^.

Recent studies have demonstrated the effectiveness of advanced materials, such as doped carbon dots and biomass-derived inhibitors, in acidic media. For instance, Rui Wan et al.^13^ reported inhibition efficiencies exceeding 97% using N, S-doped carbon dots, while Bochuan Tan et al.^14^ achieved up to 98.9% efficiency using biomass-derived carbon quantum dots. Additionally, plant extracts rich in bioactive compounds exhibit strong adsorption capabilities, forming protective films that suppress both anodic and cathodic reactions^15–18^.

Schiff base compounds containing azomethine (C = N) groups have also attracted considerable attention due to their strong adsorption tendency and excellent inhibition performance in acidic environments^19–22^. This effectiveness is attributed to heteroatoms with lone-pair electrons, which facilitate strong interaction with metal surfaces.

Despite these advances, there remains a need for efficient, low-cost corrosion inhibitors with well-understood mechanisms, particularly for aggressive environments encountered in mining-related operations. In this study, N-(1-methylpyrrolidin-2-ylidene)benzo[d]thiazol-2-amine (MPBA) was synthesized and evaluated as a corrosion inhibitor for carbon steel in 1.0 M HCl solution. Its performance was investigated using various experimental techniques over a range of temperatures.

The novelty of this work lies in the use of a deliberately synthesized, low-cost organic compound (MPBA) as an efficient corrosion inhibitor. This study provides comprehensive mechanistic insight through combined chemical, electrochemical, adsorption, and computational analyses. Density Functional Theory (DFT) and molecular dynamics (MD) simulations were employed to elucidate the interaction between MPBA molecules and the steel surface, offering a deeper understanding of the relationship between molecular structure, adsorption behavior, and inhibition performance^23–26^.

## Experimental

### Materials and metal specimens

The working electrode (WE) used for electrochemical measurements, as well as the specimens employed for gravimetric and gasometric studies, were prepared from carbon steel (C-steel) with the chemical composition listed in Table 1. All samples were cut into identical dimensions and surface areas. Before each experiment, the specimens were mechanically polished using successive grades of silicon carbide abrasive papers, starting from coarse to finer grades, to obtain a smooth and uniform surface, as previously reported^27^.

The chemicals required for the synthesis of the inhibitor, namely 1-methylpyrrolidin-2-one and benzo[d]thiazol-2-amine, were purchased from Meric Chemicals. All reagents were of analytical grade, with a stated purity exceeding 99%, and were used without further purification. Ethanol was employed as the solvent.

**Table 1:** Fourier-transform infrared spectroscopy analysis of C-steel.

Element	Mn	C	Si	S	*P*	Fe
Chemical composition, wt%	0.60	0.20	0.39	0.03	0.03	98.75

### Synthesis of the MPBA

The objective of the preparation of the compound *N*-(1-methylpyrrolidin-2-ylidene) benzo[d]thiazol-2-amine, MPBA, is to examine the protection of C-steel from destruction processes in HCl solution. The synthesis process was performed by stirring a solution of 0.01 mol of benzothiazole (1)and 0.01 mol of N-methyl pyrrolidine (2) for 1 h, followed by heating under reflux for 1 day. The precipitate obtained upon concentration was collected by filtration and crystallized from ethanol to provide the secondary amine (5), as red crystals.

**Fig. 1:**
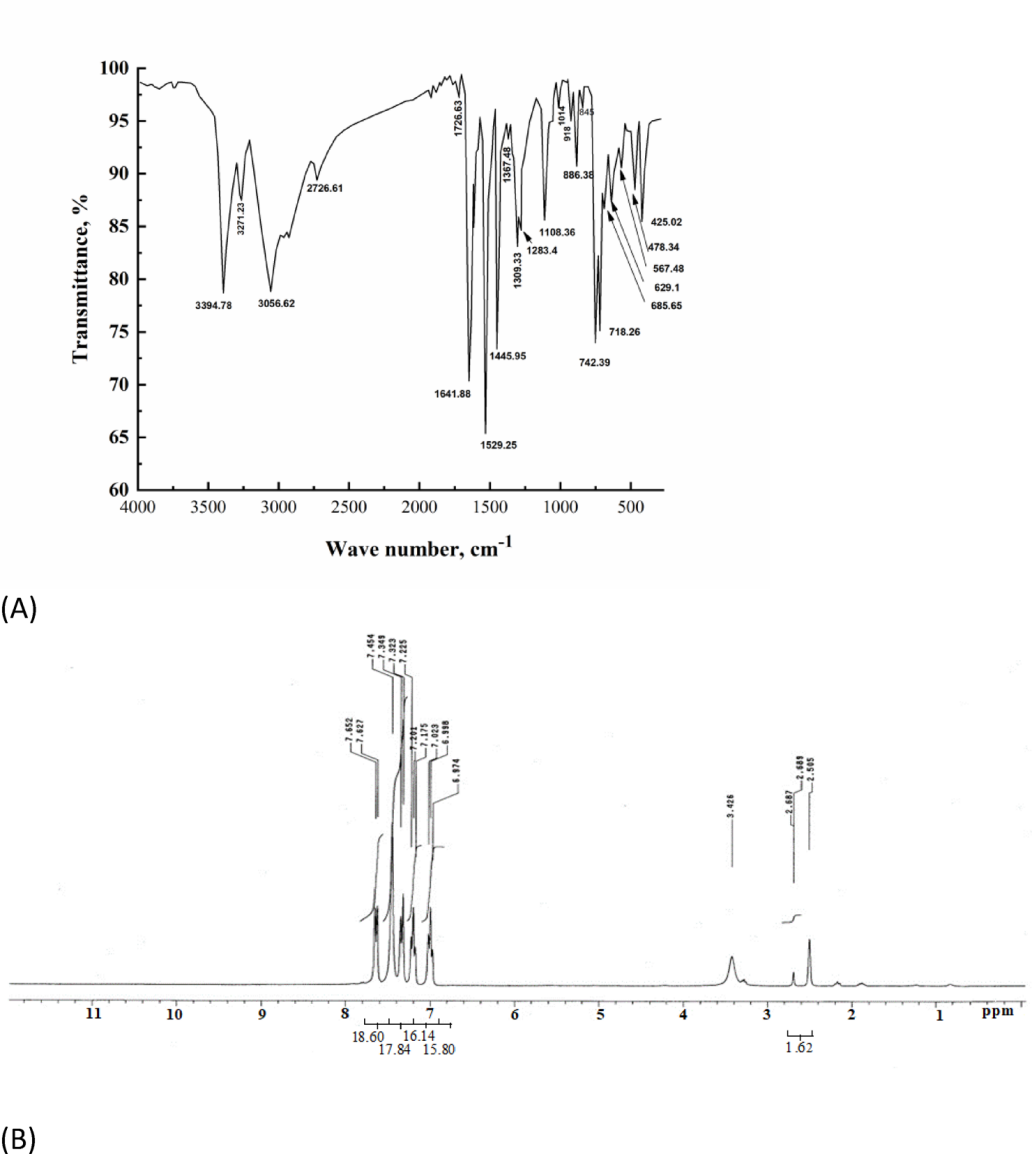
(**A**) FTIR spectrum and (**B**) 1HNMR spectrum of the MPBA compound.

The molecular structure of the freshly prepared MPBA compound was confirmed by FTIR (Fig. 1A) and mass spectroscopy (Fig. 1B).

## Results and discussion

### Structure assurance of MPBA

**Scheme 1:**
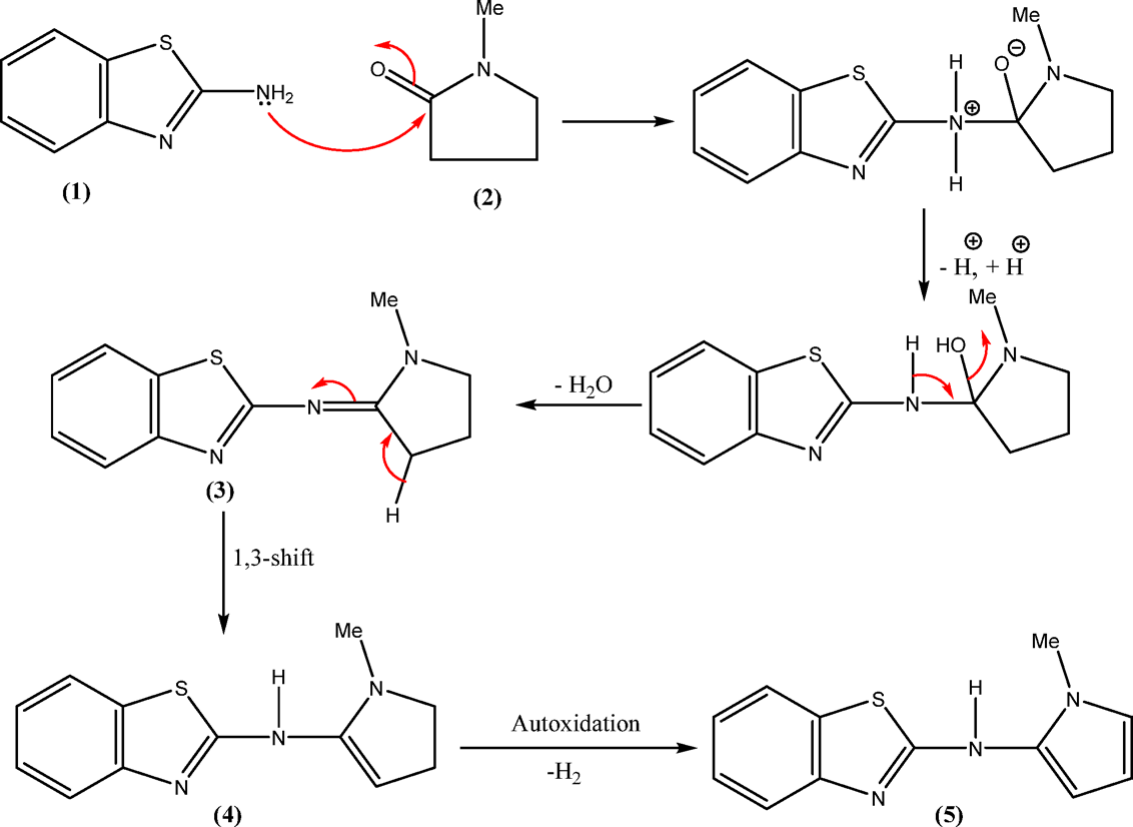
Novel synthesis of N-(1-methylpyrolidine-2-ylidine)benzo[d]thiazol-2-amine,MPBA inhibitor.

The imino compound 5 (in scheme 1) (*N*-(1-methylpyrrolidin-2-ylidene) benzo[d]thiazol-2-amine, MPBA) under physical study was obtained as a result of nucleophilic attack of aminothiazole by the electrophilic center of the exocyclic carbonyl carbon of *N*-methylpyroldenone via loss of a molecule of water, followed by 1,3-sigmatropic shift and subsequent thermal oxidation by evolution of H_2_ to attain aromaticity (Scheme 1). The spectral analysis of IR showed the presence of NH at 3394 cm^− 1^ and the lack of the C = O function, (1309, CH_3_), (1641.88, C = N), (1529, C = C).

while the ^1^HNMR showed the aromatic protons of benzo and pyrrole rings were observed in the region 7.65–7.20 ppm (m, 7 H’^S^, Ar H’^S^), 3–4.2.2 (br δ, 1H, NH), 2.88 (δ, 3 H, CH_3_),

### Mass loss, ML, investigation

The carbon steel specimens used for mass loss (ML) measurements were prepared according to a previously reported procedure^27^. Each cleaned and dried specimen was accurately weighed using a high-precision analytical balance (± 0.0001 g) to obtain the initial mass (W₁). The samples were then immersed in 250 mL of 1.0 M HCl solution, in the absence and presence of MPBA inhibitor at concentrations ranging from 5 × 10⁻⁵ M to 5 × 10⁻³ M, for 8 h. After immersion, the specimens were removed, rinsed thoroughly, dried, and reweighed to obtain the final mass (W₂). All experiments were performed in triplicate, and the average values were reported.

The corrosion rate (*r*_g_) was calculated using the following Eqs^28,29^:

1 $$\:{r_g} = \:\frac{{\Delta \:W}}{{At}}\:$$

where ∆W = W_1_ – W_2_, *t* is the immersion time measured in hours, and A is the contact area of the metallic surface. Such values of *r*_g_ are utilized to deduce the surface coverage, *θ*, and the percentage of inhibition efficiency (*η*_w_) of the MPBA inhibitor via the Eqs^10,29^:

2 $$\:\:\theta \: = \left( {1 - \frac{{{r_g}}}{{{r^ \circ }_c}}} \right)$$

3 $$\:\:\eta {\:_w}\: = \left( {1 - \frac{{{r_g}}}{{{r^ \circ }_g}}} \right)100$$

where *r*_g_^º^ and *r*_g_ are the rates of metallic destruction in HCl electrolytes without and with the MPBA inhibitor, respectively.

### Gasometry study, GS

The gasometry (GS) experiments were carried out using procedures similar to those employed in the mass loss measurements, and the carbon steel specimens were prepared in the same manner^27^. The cleaned and dried carbon steel sample was immersed in 250 mL of 1.0 M HCl solution in the absence and presence of MPBA inhibitor at concentrations ranging from 5 × 10⁻⁵ M to 5 × 10⁻³ M. After an initial induction period, hydrogen evolution began, and the volume of H₂ gas produced was collected over water as a function of immersion time. The experimental setup and reaction vessel used for hydrogen collection have been described in detail as shown previous studies^29–31^. Each experiment was performed in triplicate, and the average values were reported.

The data of the volume of the produced H_2_, *V*, at various immersion times, *t*, are used to determine the rate of the metal destruction, *r*_H_. The values of *θ* and *η*_H_ can be computed, successively, from the *r*_H_ values according to the following relations^29,32^:

4 $$\:\:\theta \: = \left( {1 - \frac{{{r_H}}}{{{r^ \circ }_H}}} \right)100$$

5 $$\:\:\eta {\:_H}\: = \left( {1 - \frac{{{r_H}}}{{{r^ \circ }_H}}} \right)100$$

where *r*ᵒ_H_ and *r*_H_ are the destruction reaction rates in hydrochloric acid electrolytes without and with the MPBA, respectively.

### Electrochemical study

Potentiodynamic polarization (PDP) and electrochemical impedance spectroscopy (EIS) measurements were employed to evaluate the inhibitive performance of MPBA toward carbon steel corrosion in 1.0 M HCl solution^27^. All electrochemical experiments were conducted using a conventional three-electrode system in a double-walled silica glass cell (250 mL). Carbon steel served as the working electrode (WE), a platinum wire as the counter electrode, and a saturated calomel electrode (SCE) as the reference electrode.

The experiments were performed in the absence and presence of MPBA at concentrations ranging from 5 × 10⁻⁵ M to 5 × 10⁻³ M. Before each measurement, the working electrode surface was mechanically polished using successive grades of emery papers down to a fine grit, following a previously reported procedure^27^, then rinsed thoroughly with distilled water and dried. All measurements were carried out under unstirred conditions at 25 °C.

Before each electrochemical measurement, the working electrode was immersed in the test solution and allowed to stabilize at the open circuit potential (OCP) for approximately 40 min, or until a steady-state potential was reached.

Potentiodynamic polarization curves were recorded by sweeping the working electrode potential at a scan rate of 0.2 mV s⁻¹ over the potential range of − 850 to − 350 mV versus SCE around the corrosion potential (*E*_corr_) at 25 °C. All electrochemical measurements were carried out using a VoltaLab PGZ-301 potentiostat.

The corrosion current density in the corrosive solution free of inhibitor, $\:{i}_{\mathrm{c}\mathrm{o}\mathrm{r}\mathrm{r}}^{^\circ\:}$, and in the presence of the MPBA inhibitor, *i*_corr_ can be computed by extrapolation of anodic and cathodic Tafel lines to *E*_corr_. The values of *θ* and percent of protection (*η*_p_) were deduced, successively, from the following Eqs^32–34^:

6 $$\:\:\theta \: = \left( {1 - \frac{{{i_{corr}}}}{{{i^ \circ }_{corr}}}} \right)$$

7 $$\:\:{\eta _p}\left( {1 - \frac{{{i_{corr}}}}{{{i^ \circ }_{corr}}}} \right)100$$

The EIS study was done on carbon steel electrodes in corrosive and inhibitive solutions at frequencies varied between 100 and 5 × 10^− 6^ kHz with an amplitude of 0.01 V. The acquired EIS data were fitted to suitable electrical equivalent circuit diagrams (EECDs) using licensed ZSimpWin software^35^.

The$\:{R}_{\mathrm{c}\mathrm{t}}^{^\circ\:}$ and $\:{R}_{c\mathrm{t}}\:$which represents the charge transfer resistance for the inhibitor-free acid electrolytes without and with the MPBA Schiff base are utilized to compute the values of *θ*, and the percent of inhibition (*η*_I_) utilizing Eqs. 8 and 9 successively^34,36^:

8 $$\:\:\theta= \left( {1 - \frac{{{R^ \circ }_{ct}}}{{{R_{ct}}}}} \right)100$$

9 $${\eta _I} = \left( {1 - \frac{{{R^ \circ }_{ct}}}{{{R_{ct}}}}} \right)100$$

Utilizing $\:\omega\:$_max_ = 2π*f*_max_, the double-layer capacitance, *C*_dl_can be deduced via the Eqs^37,38^.

10 $${C_{dl}} = Y \circ {\left( {{\omega _{\max }}} \right)^{n - 1}}$$

### Surface analysis

The surface morphology of corroded and uncorroded carbon steel coupons in the investigated solutions was examined using a scanning electron microscope (SEM). Three samples were analyzed: a clean and dry carbon steel specimen, a specimen immersed in dilute HCl solution, and another specimen immersed in a hydrochloric acid solution containing 0.005 M MPBA at 25 °C. After four hours of immersion, the samples were removed from the solutions, thoroughly dried, and then examined under SEM.

### Theoretical investigations

The computations of the DFT were conducted utilizing Gaussian 09 software^39^ to evaluate the behavior of the MPBA compound as a corrosion inhibitor. These computations included geometry optimizations of the inhibitors using the B3LYP method^40^, which combines Becke’s three-parameter functional (B3) with the gradient-corrected correlation LYP functional^41^. The 6-31G (d, p) basis set was applied for all atoms in the calculations.

The application of theoretical calculations in the design and evaluation of corrosion inhibitors has been significantly advanced through the development of Density Functional Theory (DFT)^42^. This approach enables a detailed understanding of dispersive, repulsive, and electrostatic interactions between solute and solvent molecules, particularly when combined with the conductor-like polarizable continuum model (CPCM) for solvation. DFT provides a robust theoretical framework for calculating global reactivity descriptors that quantitatively reflect the intrinsic chemical activity of inhibitor molecules. These descriptors include the energies of the highest occupied molecular orbital (*E*_HUMO_), the lowest unoccupied molecular orbital (*E*_LUMO_), and the energy gap (ΔE), which are widely used to assess corrosion inhibition efficiency.

From a practical perspective, DFT calculations allow meaningful correlations with experimental results obtained in aqueous environments. They offer valuable insights into the electronic structure of inhibitor molecules, their adsorption behavior on metal surfaces, and the nature of inhibitor–metal interactions, thereby contributing to the rational optimization of corrosion protection performance.

Global hardness ($\:\eta\:)$, softness ($\:\sigma\:)$, and the number of electrons transferred (N) can be computed by the relations:

11 $$\:\eta\:=\frac{{E}_{LUMO}-{E}_{HOMO}}{2}$$

12 $$\:\:\:\:\:\:\:\:\:\:\:\:\:\:\:\:\:\:\:\:\:\:\:\:\:\:\:\:\:\:\:\:\sigma\:=\frac{1}{\eta\:}$$

13 $$\:\:\:\:\:\:\:\:\:\:\:\:\:\:\:\:\:\:\:\:\:\:\:\:\:\:\:\:\:\:\:{\Delta\:}{N}_{110}=\frac{\varphi\:-{\chi\:}_{inh}}{2({\eta\:}_{Fe}+{\eta\:}_{inh})}\:\:$$

where $\:\varphi\:$,$\:\:{\chi\:}_{inh}$,$\:\:{\eta\:}_{Fe}$, and $\:{\eta\:}_{inh}$ are the work function of Fe(110) (4.82 eV)^43^, the electronegativity of the organic substance (MPBA), the chemical hardness of iron, and the MPBA compound, successively.

### Monte Carlo (MC) and molecular dynamics (MD) simulations

The adsorption behavior of the organic compound MPBA on the iron surface was investigated using Monte Carlo simulations implemented in the Adsorption Locator module of Materials Studio. Molecular dynamics (MD) simulations were subsequently performed using the Forcite module in Materials Studio 17. All MD simulations were conducted in the canonical (NVT) ensemble employing an Andersen thermostat, with a time step of 1.0 fs and a total simulation time of 20 ps at 298 K.

The iron surface was modeled by cleaving the Fe crystal along the (110) plane, and a five-layer slab was constructed. The Fe (110) surface was expanded into a (10 × 10) supercell, with a vacuum layer of 25 Å introduced above the surface. This region was filled with 100 water molecules and a single MPBA inhibitor molecule to simulate the aqueous environment. The COMPASS force field was employed for geometry optimization and system initialization. Electrostatic interactions were calculated using the Ewald summation method with a high precision of 1 × 10⁻⁵ kcal mol⁻¹^44^.

The adsorption energy (*E*_ads_) of the MPBA molecule on the iron surface was computed to evaluate the inhibitor’s adsorption strength and effectiveness. In addition, radial distribution function (RDF) analysis was performed to examine the nature of interactions and potential chemical bonding between MPBA molecules and iron atoms.

## Results and discussion

### Mass loss, ML, investigation

**Fig. 2:**
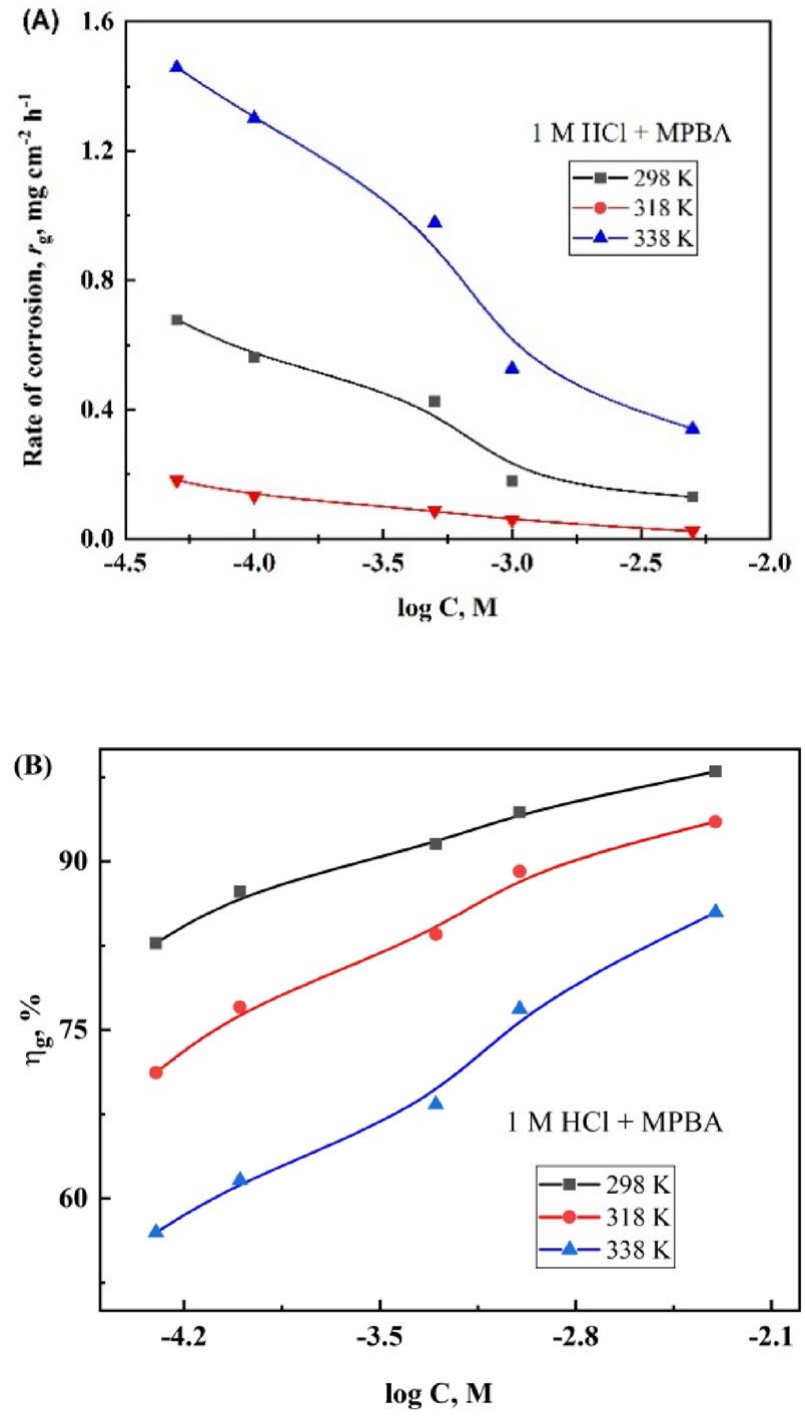
(**A**) Variation of the corrosion rate (*r*_g_) of carbon steel at 1.0 M HCl and (**B**) inhibition efficiency (*η*_w_%) as a function of log C of the MPBA compound.

The mass loss (*ML*) method was employed to investigate both the corrosive effect of HCl on carbon steel and the protective performance of the MPBA compound at various temperatures. The measured corrosion rates (*r*_g_) were used to calculate the corresponding surface coverage (*θ*) and inhibition efficiency (*η*_w_), as presented in Table 2. In the absence of the inhibitor, *r*_g_ was 1.0494 ± 0.01 mg cm⁻² h⁻¹, which decreased to 0.0243 ± 0.01 mg cm⁻² h⁻¹ in the presence of 5 mM MPBA at 25 °C. Figure 2A shows that *r*_g_ decreases with increasing MPBA concentration at all investigated temperatures. Increasing the MPBA content in the aggressive medium further reduces *r*_g_ and correspondingly increases *η*_w_ (Table 2; Fig. 2A&B). The sigmoidal shape of these plots confirms the strong adsorption of MPBA molecules on the steel surface, which effectively protects the metal from attack by corrosive Cl⁻ ions^37,45^. The slight decrease in *θ* and *η*_w_ with increasing temperature can be attributed to the partial desorption of MPBA molecules from the metal surface into the bulk solution. This desorption process supports a predominantly physisorption-controlled adsorption mechanism^46^.

**Table 2:** Corrosion rate (*r*_g_, mg cm⁻² h⁻¹), surface coverage (*θ*), and inhibition efficiency (*η*_w_) of MPBA for carbon steel in 1.0 M HCl at different temperatures, obtained from gravimetric measurements. Values are reported as mean ± γ, where γ denotes the standard deviation.

Conc. mM	298 K			313 K			338 K		
	r_g_, *±*ɤ mg cm^− 2^ h^− 1^	θ	η_w_ (%)	r_g_, *±* ɤ mg cm^− 2^ h^− 1^	θ	η_w_ (%)	r_g_, *±* ɤ mg cm^− 2^ h^− 1^	θ	η_w_ (%)
0.00	1.0494 *±* 0.01	-	-	2.0051 *±* 0.02	-	-	3.3874 *±* 0.03	-	-
0.05	0.1811 *±* 0.02	0.83	82.74	0.6779 *±* 0.01	0.66	66.19	1.4578 *±* 0.02	0.57	56.97
0.10	0.1333 *±* 0.02	0.87	87.30	0.5620 *±* 0.02	0.72	71.97	1.3008 *±* 0.02	0.62	61.60
0.50	0.0884 *±* 0.03	0.92	91.57	0.4257 *±* 0.02	0.79	78.77	0.9784 *±* 0.01	0.71	71.12
1.00	0.0587 *±* 0.01	0.94	94.40	0.1791 *±* 0.03	0.91	91.07	0.5271 *±* 0.03	0.84	84.44
5.00	0.0243 *±* 0.02	0.98	97.68	0.1297 *±* 0.03	0.94	93.53	0.3410 *±* 0.02	0.90	89.93

### Gasometric study, GS

**Fig. 3:**
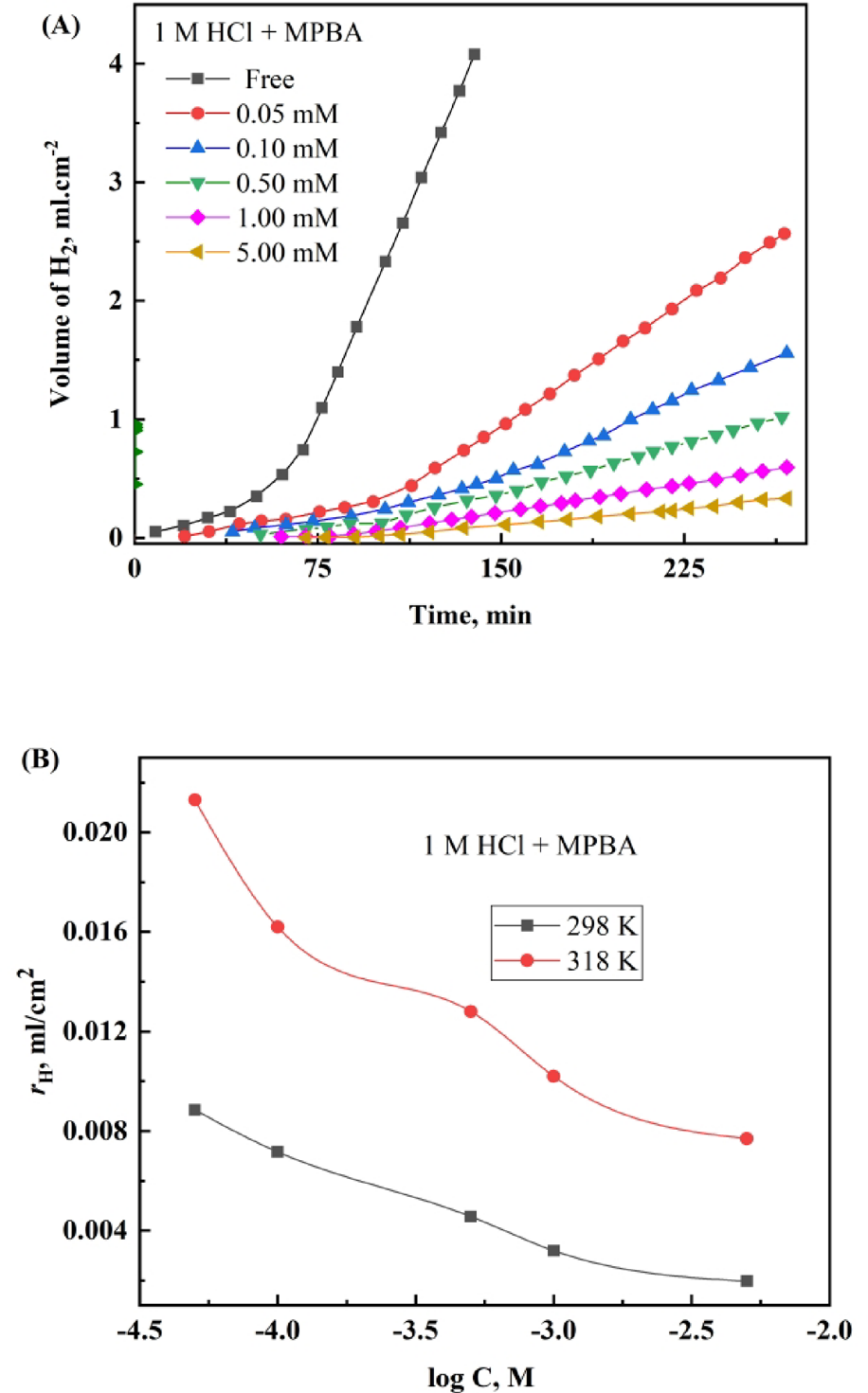
(**A**) V-time curves of C-steel in 1.0 M HCl free of and mixed with various additives of MPBA and (**B**) the *r*_g_ with the log C of MPBA in 1.0 M HCl, respectively.

The gasometric technique was employed to investigate the inhibitory effect of MPBA on the corrosion of carbon steel and the associated hydrogen evolution. In 1.0 M HCl, the volume of hydrogen evolved (*V*_H_) increased after an induction period (τ) of approximately 40 min, followed by a rapid rise with longer immersion times. Adding MPBA to the acidic solution significantly extended the induction period, with τ reaching 100 and 165 min at 0.05 mM and 5 mM MPBA, respectively. Higher temperatures, however, reduced τ. Figure 3A shows the variation of *V*_H_ with immersion time for carbon steel in HCl in the absence and presence of different MPBA concentrations. Figure 3B illustrates the relationship between the hydrogen evolution rate (*r*_H_) and the logarithm of MPBA concentration. The sigmoidal (S-shaped) curves confirm the adsorption of MPBA molecules onto the carbon steel surface^47^.

The accumulation of H₂ on the carbon steel surface over time reflects the breakdown of the oxide layer by Cl⁻ ions in the solution^29,47,48^. The continuous increase in *V*_H_ with immersion time indicates ongoing corrosion of the bare metal^29^. The observed extension of the induction period (τ) at higher MPBA concentrations suggests a delay in the corrosion process due to inhibitor adsorption^28^. The hydrogen evolution rate (*r*_H_) was determined from the slope of the linear portions of the *V*_H_ versus log t plots (Fig. 3A). Figure 3B presents the sigmoidal relationship between *r*_H_ and log C of MPBA at 25 °C and 45 °C. This S-shaped behavior, like that observed in the mass loss measurements, confirms the adsorption of MPBA molecules on the carbon steel surface^47^.

Although the surface coverage (*θ*) and inhibition efficiency (*η*_H_%) increase with increasing MPBA concentration, both parameters decrease with rising temperature (*T*), as shown in Table 3. This behavior suggests the partial desorption of adsorbed MPBA molecules from the metal surface into the bulk solution, thereby supporting a predominantly physisorption-controlled inhibition mechanism^49^.

**Table 3:** Corrosion rate (*r*_g_, mL cm⁻² h⁻¹), surface coverage (θ), and inhibition efficiency (*η*_H_) of MPBA for carbon steel in 1.0 M HCl at 298 and 318 K, as determined by gasometric measurements. (Values in parentheses correspond to measurements at 318 K).

Conc. (mM)	r_H_, mL/cm^2^/min *±* ɤ	θ	η_H_
0.00	0.04760 *±* 0.01 [0.0871 *±* 0.02]^*^	---	---
0.05	0.00885 *±* 0.02 [0.0213 *±* 0.02]	0.814 [0.756]	81.4 [75.6]
0.10	0.00715 *±* 0.02 [0.0162 *±* 0.02]	0.850 [0.814]	85.0 [81.4]
0.50	0.00457 *±* 0.01 [0.0128 *±* 0.01]	0.904 [0.853]	90.4 [85.3]
1.00	0.00321 *±* 0.01 [0.0102 *±* 0.01]	0.933 [0.883]	93.3 [88.3]
5.00	0.00197 *±* 0.01 [0.0077 *±* 0.01]	0.959 [0.912]	95.9 [91.2]

**Fig. 4:**
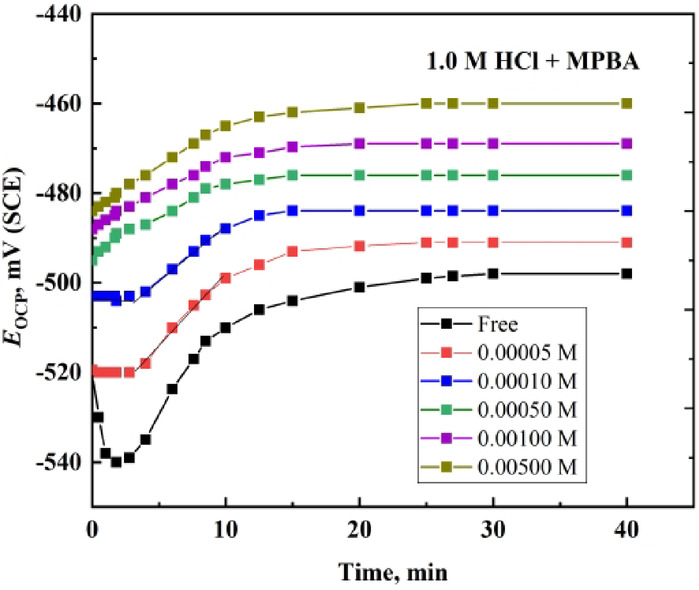
Open-circuit potential (OCP) of carbon steel in 1.0 M HCl in the presence of different concentrations of the MPBA compound.

### Open circuit potential

The variation of the open-circuit potential (OCP) with immersion time for carbon steel in 1.0 M HCl is illustrated in Fig. 4. It is observed that the steady state potential (*E*_st_) is attained within a time interval ranging from 20 to 30 min. For the uninhibited acid solution, the estimated potential reaches − 0.498 V. Upon the addition of the MPBA inhibitor, *E*_s_ shifts toward more positive values, increasing from − 0.468 V at a concentration of 1 × 10⁻^3^ M to − 0.459 V at 5 × 10⁻³ M MPBA in 1.0 M HCl. This positive displacement of *E*_s_ is attributed to the rupture of the air-formed oxide layer generated before immersion, while the subsequent stabilization of the potential promotes further thickening of the oxide film^28,50^. Moreover, the slight positive variation in potential observed over time for the inhibited solutions, compared to the blank solution, can be ascribed to the formation of corrosion products and/or the adsorption of inhibitor molecules onto the metal surface.

### Potentiodynamic study, *PD*

**Fig. 5:**
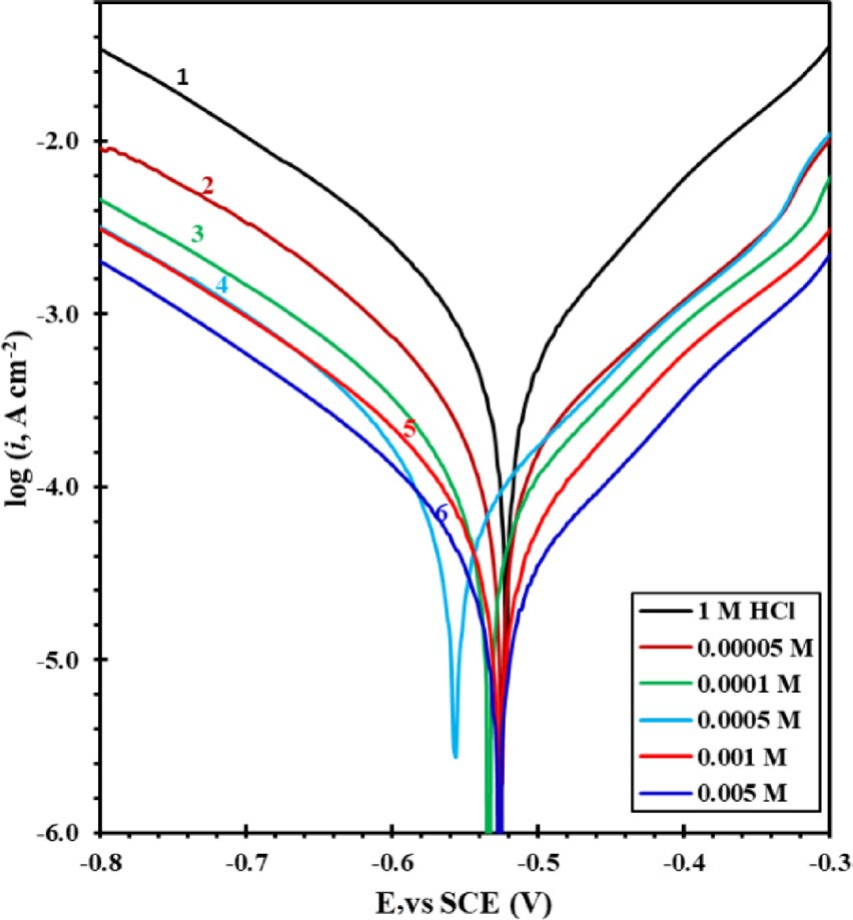
The potentiodynamic polarization curves of C-steel in 1.0 M HCl solution, free of and mixed with various amounts of MPBA compound, at 298 K.

The potentiodynamic polarization (*PD)* curves of carbon steel in both the aggressive and inhibitive (MPBA) acid solutions are shown in Fig. 5. The main electrochemical corrosion factors, namely the corrosion potential, *E*_corr_, *R*_p_, *i*_corr_, *β*_c_, *β*_a_, *θ*, and *η*_p_ were calculated and are summarized in Table 4. The corrosion current density in the uninhibited HCl solution was found to be 1.07 mA.cm^− 2^ and decreased progressively with increasing concentration of the MPBA compound. Notably, *i*_corr_ was reduced to 0.04 mA.cm^− 2^ in the presence of 5 mM MPBA. The values *β*_a_ and *β*_c_ exhibited no significant variation, indicating that the corrosion mechanism remains essentially unchanged in both the free acid and inhibited solutions^51,52^.

In addition, Fig. 5 indicates that the presence of MPBA inhibitor does not exert a significant influence on the corrosion potential (*E*_corr_) compared with that observed in the uninhibited HCl solution. This behavior suggests that the MPBA compound simultaneously retards both the anodic dissolution of iron and the cathodic reduction of hydrogen ions. The variation in Δ*E*_corr_, defined as the difference between *E*_corr_ values in the uninhibited and inhibited solutions, is approximately *±* 33 mV, confirming that the MPBA compound acts as a mixed-type inhibitor^46,52^.

The inhibition efficiency (*η*_p_) of MPBA was calculated from the corrosion current density (*i*_corr_) values obtained in the absence and presence of the MPBA inhibitor using Eq. (6). The inhibition efficiency increases with increasing MPBA concentration as a result of the progressive decrease in *i*_corr_ value. This behavior demonstrates that MPBA effectively protects both the anodic sites associated with iron dissolution and the cathodic active sites responsible for hydrogen evolution on the carbon steel surface^53^.

The protection efficiency (*η*ₚ) of the MPBA inhibitor was calculated from the corrosion current density (*i*_corr_) values obtained in the aggressive HCl solution in the absence and presence of the MPBA Schiff base using Eq. (6). Protection efficiency increased progressively with increasing MPBA concentration, which is attributed to the marked reduction in *i*_corr_. This behavior indicates that the MPBA inhibitor effectively suppresses both the anodic dissolution of iron and the cathodic hydrogen evolution reactions on the investigated carbon steel surface^53^.

The addition of the MPBA Schiff base inhibitor to the corrosive solution raises the *R*_p_ value from 42 Ω cm^2^ in the corrosive dilute hydrochloric acid solution to 449 Ω cm^2^ when 5 mM of the MPBA compound is mixed with the corrosive solution, proving the uniformity of the adsorbed protective layer formed by the adsorption process. The rise in the *R*_p_ and *ɳ*_p_ values at higher additions of the MPBA inhibitor could prove the growth of an adsorbed layer of MPBA molecules on the examined carbon steel^53^.

**Table 4:** Potentiodynamic polarization parameters calculated for C-steel in 1.0 M HCl, free of and mixed with various additions of the MPBA compound, at 298 K. (ɤ represents the standard deviation values).

Conc., M	E_corr_, mV, SCE	i_corr_ *±* ɤ, mA cm^− 2^	Β_a_, mV dec^− 1^	Β_c_, mV dec^− 1^	Rp, Ω cm^2^	η_*p*_, %
0.00	−524	1.0697 *±* 0.05	153	−179	42	-
0.05	−504	0.1982 *±* 0.03	155	−174	158	81.47
0.10	−500	0.1691 *±* 0.04	166	−189	191	84.19
0.50	−503	0.0998 *±* 0.02	158	−167	204	90.67
1.00	−533	0.0687 *±* 0.03	162	−166	283	93.58
5.00	−522	0.0412 *±* 0.01	138	−148	449	96.15

### The impedance, EIS study

**Fig. 6:**
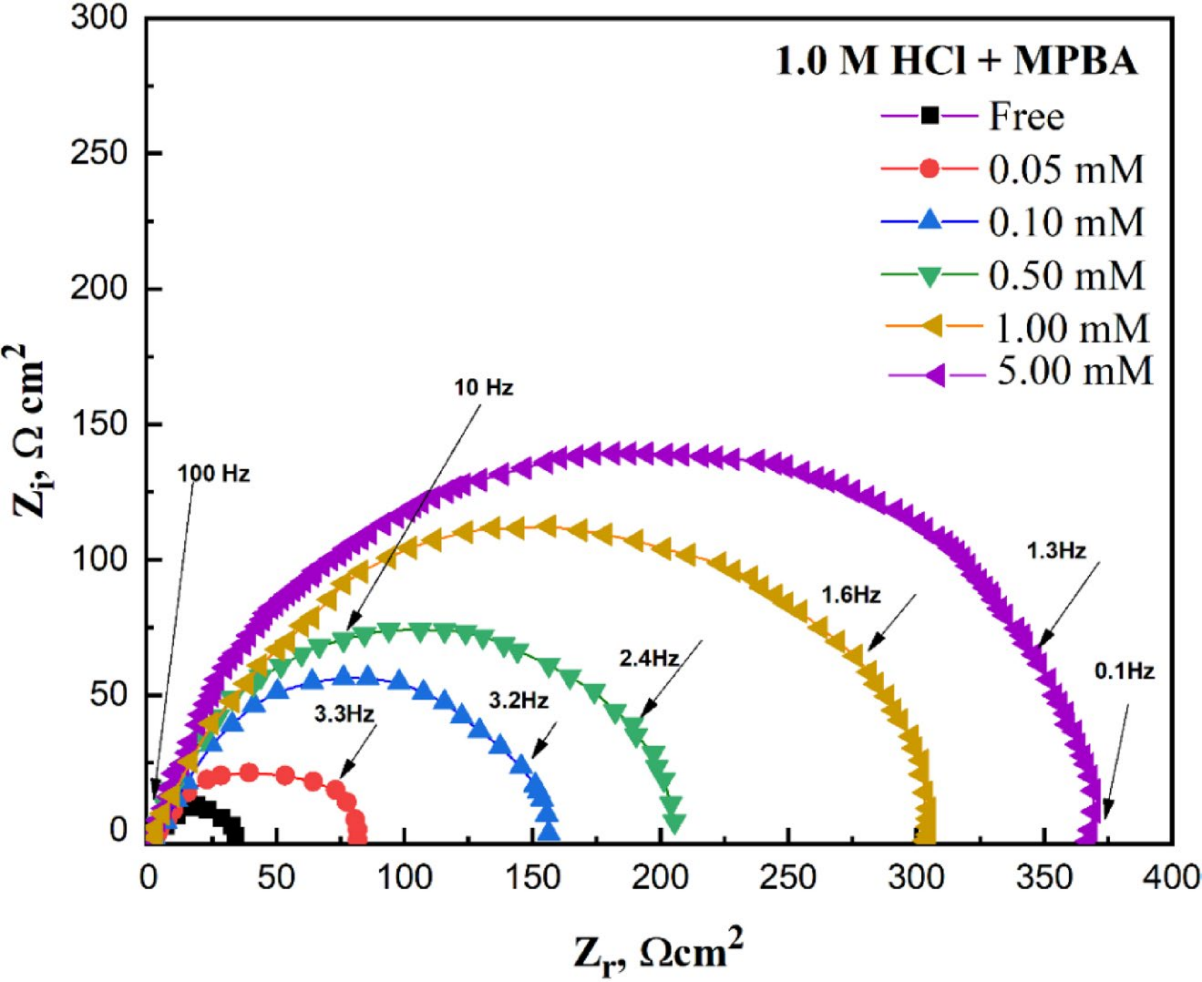
The Nyquist plots of carbon steel in 1.0 M HCl solution, free of and mixed with various additions of MPBA compound, at 298 K.

**Fig. 7:**
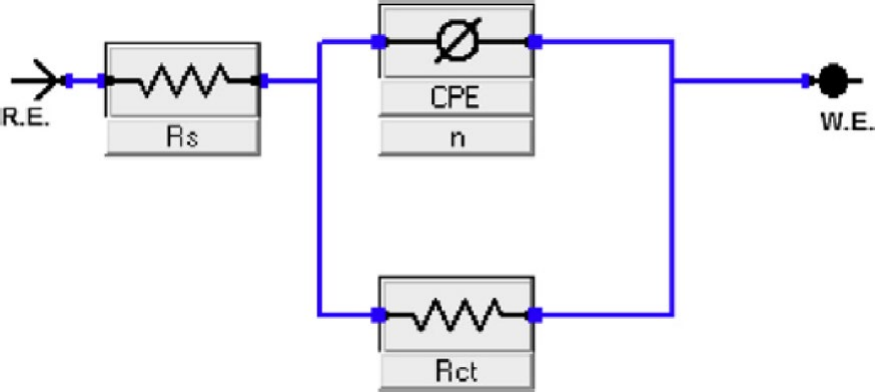
An equivalent circuit model was used to fit the EIS data in the absence and presence of the DMAB compound.

Electrochemical impedance spectroscopy (EIS) is a rapid, non-destructive technique used to analyze the Nyquist response of carbon steel in corrosive acidic media, both in the absence and presence of MPBA (Fig. 6). The addition of MPBA to the HCl solution significantly modifies the impedance behavior, reflecting changes in the electrochemical processes at the metal/solution interface. The experimental data are satisfactorily fitted using the equivalent electrical circuit shown in Fig. 7^54^.

The equivalent circuit consists of the solution resistance (Rₛ), charge-transfer resistance *R*_ct_, and a constant phase element (CPE) that accounts for the non-ideal behavior of the electrical double layer. The *R*_ct_ value reflects the corrosion resistance and increases with inhibitor adsorption, while the CPE describes surface heterogeneity and deviations from ideal capacitive behavior. The impedance of the CPE is expressed as^55,56^:

14 $$\:{Z}_{\mathrm{C}\mathrm{P}\mathrm{E}}=\frac{1}{Q(j\omega\:{)}^{n}}\:\:\:\:\:$$

where $\:Q$ is the CPE magnitude, $\:\omega\:$ is the angular frequency, $\:j$ is the imaginary unit, and $\:n$ (0 ≤ *n* ≤ 1) represents the deviation from ideal capacitive behavior.

The Nyquist plots (Fig. 6) exhibit a single depressed semicircular capacitive loop, indicating that a charge-transfer mechanism predominantly controls the corrosion process. The deviation from ideal semicircular behavior is attributed to surface heterogeneity and non-uniform current distribution. Moreover, the presence of a single time constant suggests a dominant interfacial process over the frequency range investigated.

The formation of capacitive loops in both uninhibited and inhibited solutions indicates the development of an interfacial layer on the carbon steel surface. In the presence of higher concentrations of MPBA, this layer becomes more protective due to the adsorption of inhibitor molecules, which form a compact film that limits the interaction between the aggressive HCl medium and the metal surface^57–59^. Increasing the MPBA concentration results in a progressive enlargement of the semicircle diameter, corresponding to higher charge-transfer resistance with improving corrosion protection.

**Fig. 8:**
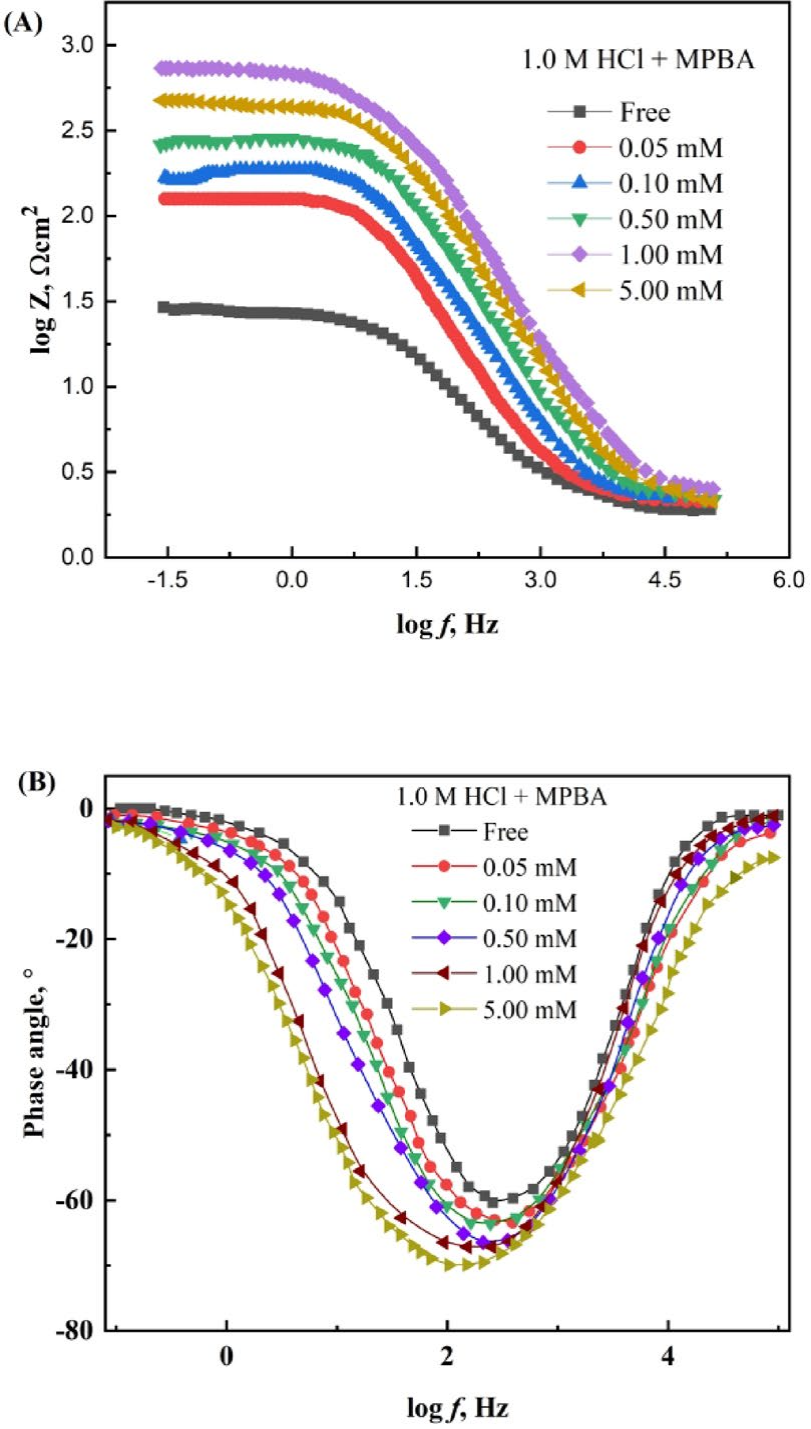
(**A**) The Bode and (**B**) the phase angle plots of C-steel in 1 M HCl solutions free of and mixed with various additions of MPBA compound, successively, at 25 °C.

The Bode magnitude and phase angle plots were used to evaluate the inhibitive action of MPBA on carbon steel in HCl, as shown in Fig. 8A and B, respectively. The magnitude plots display capacitive behavior at intermediate frequencies and resistive responses at both high and low frequencies, characteristic of a charge-transfer-controlled corrosion process.

The phase angle increases progressively with MPBA concentration, indicating enhanced capacitive behavior due to the adsorption of inhibitor molecules on the steel surface. A maximum value of approximately 70° is observed at 5 mM MPBA, reflecting improved surface protection. This increase also suggests the formation and stabilization of a protective adsorbed film, which contributes to the rise in inhibition efficiency (*η*_I_) with inhibitor concentration^22,60^.

Moreover, the shift in the phase angle toward more negative values at higher concentrations supports the formation of a compact barrier layer that effectively limits interaction between the aggressive HCl medium and the carbon steel surface^22,61,62^.

The electrochemical impedance parameters, including charge-transfer resistance (*R*_ct_), double-layer capacitance (*C*_dl_), and inhibition efficiency (*η*_I_), were obtained from fitted data and are summarized in Table 5. The increase in *R*_ct_ with MPBA concentration indicates enhanced adsorption of inhibitor molecules on the carbon steel surface, leading to a reduced corrosion rate^22^.

In contrast, the constant phase element (CPE) magnitude (Q) decreases in the presence of MPBA, suggesting a reduction in the local dielectric constant and/or an increase in the thickness of the electrical double layer. This behavior is consistent with the replacement of adsorbed water molecules by MPBA, resulting in the formation of a more compact protective film^53^.

Similarly, the decrease in *C*_dl_ with increasing inhibitor concentration reflects changes in interfacial properties, supporting the formation of an adsorbed layer that effectively suppresses metal dissolution^22,63^.

**Table 5:** The EIS data of C-steel in 1.0 M HCl free of and mixed with various additions of the MPBA compound, at 298 K.

Conc., M	*R*_s_, Ω cm^2^	Q_dl_, 10^4^, Ω^−1^s^*n*^ cm^− 2^	*n* %	Error of *n*	*R*_ct_, +SD Ω cm^2^	C_dl,_ µF cm^− 2^	η_I_ %
0.00	2.32	0.0002215	0.83	1.22	25.4 *±* 0.12	280.4	-
5 × 10^− 5^	2.40	0.0001745	0.85	1.04	132 *±* 0.14	89.3	80.8
1 × 10^− 4^	2.60	0.0001585	0.74	1.12	174 *±* 0.15	70.0	85.4
5 × 10^− 4^	2.30	0.0001366	0.80	0.83	249 *±* 0.13	108	89.8
1 × 10-3	2.70	0.0000656	0.70	0.50	348 *±* 0.16	75.0	92.7
5 × 10-3	3.10	0.0000210	0.70	0.50	794 *±* 0.14	73.0	96.8

### Adsorption isotherm

The inhibition of steel corrosion in acidic media by organic inhibitor molecules such as MPBA is primarily governed by the adsorption process occurring at the metal/solution interface. This adsorption behavior depends on several factors, including the presence of multiple adsorption centers within the inhibitor molecule and the temperature of the corrosive medium. The adsorption of MPBA molecules on the steel surface proceeds via a substitution mechanism, in which inhibitor molecules displace pre-adsorbed water molecules from the metal surface. This process can be represented by the following equilibrium reaction^64^.

15 $$MPBA{\rm{ }}(sol){\rm{ }} + {\rm{ }}z{\rm{ }}W{\rm{ }}(ads){\rm{ }} \leftrightarrow {\rm{ }}MPBA{\rm{ }}(ads){\rm{ }} + {\rm{ }}z{\rm{ }}W{\rm{ }}(sol){\rm{ }}$$

where $\:z\:$denotes the number of adsorbed water molecules, W_(ads)_, replaced by a single MPBA molecule upon adsorption. This substitution mechanism facilitates the formation of a protective adsorbed layer, thereby reducing metal dissolution and enhancing corrosion resistance.

### Adsorption isotherm analysis

The adsorption behavior of the MPBA inhibitor molecules on the carbon steel surface was evaluated using adsorption isotherm models to clarify the nature of the metal–inhibitor interaction. The degree of surface coverage (θ) was calculated from inhibition efficiency values obtained from electrochemical measurements, assuming θ = η_w_/100. Several adsorption isotherms were examined, including Langmuir, Temkin, and the Freundlich models.

Among the tested models, the Langmuir adsorption isotherm provided the best linear fit, as evidenced by the high correlation coefficient (R² ≈ 1). The Langmuir model is expressed as^65^:

16 $$\:\frac{C}{{\theta \:}} = \frac{1}{{{K_{ads}}}} + C\:$$

where *C* is the inhibitor concentration and $\:{K}_{\mathrm{ads}}\:$ is the adsorption–desorption equilibrium constant. The linear plots of $\:C/\theta\:\:$ versus $\:C$ yield regression coefficients ($\:{R}^{2}$) close to unity, confirming the applicability of the Langmuir model and supporting the formation of a single adsorbed inhibitor layer on the carbon steel surface^29,30^.

**Fig. 9:**
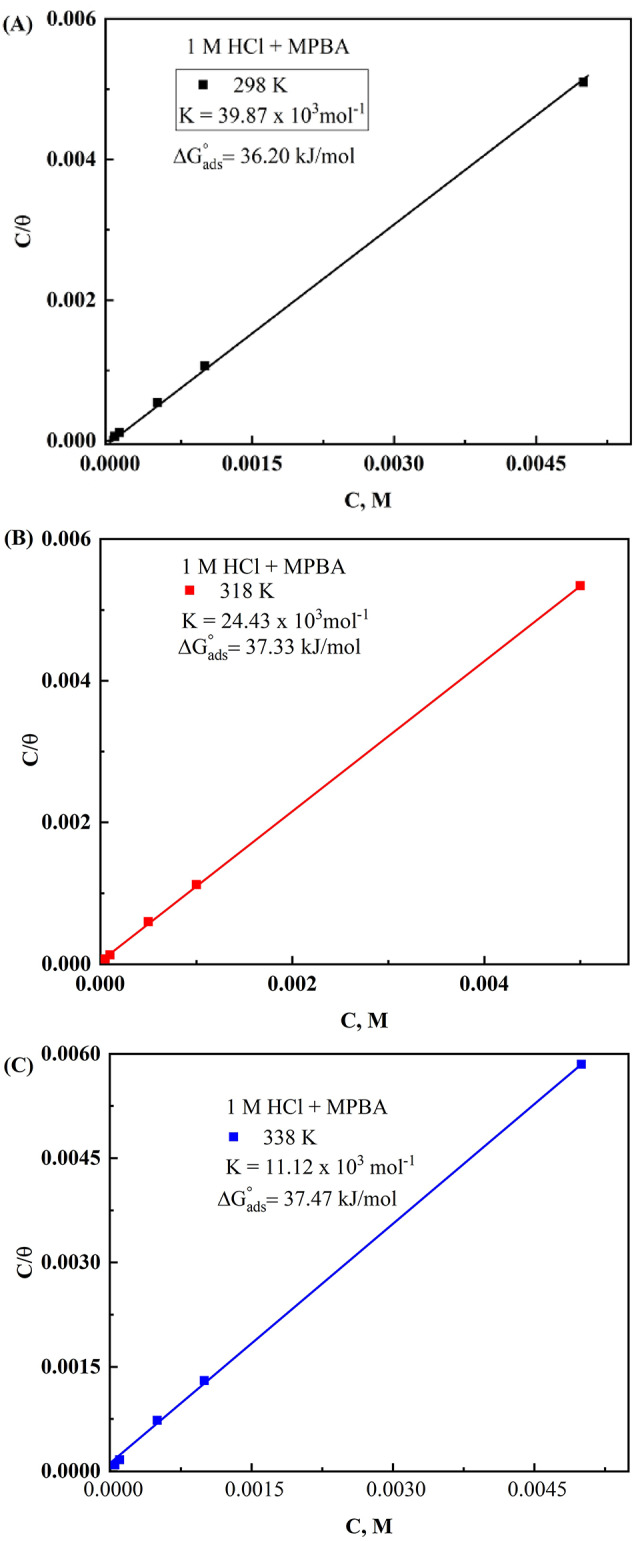
Langmuir Adsorption isotherms of the MPBA compound on the C-steel surface in 1.0 M HCl at various temperatures (**A**), (**B**), and (**C**), respectively.

Figure 9 (A–C) shows the Langmuir plots at 298, 318, and 338 K. The near-unity slopes confirm that the adsorption of MPBA on the carbon steel surface follows Langmuir behavior. The intercepts of these plots were used to determine the adsorption equilibrium constants ($\:{K}_{\mathrm{a}\mathrm{d}\mathrm{s}}$), which are listed in Table 6. The observed decrease in the equilibrium constant with increasing temperature indicates partial desorption of MPBA molecules from the metal surface at higher temperatures.

The value of $\:{K}_{\mathrm{a}\mathrm{d}\mathrm{s}}$, reflects the strength of the adsorption process, and the standard Gibbs free energy of adsorption ($\:{\Delta\:}{G}_{\mathrm{ads}}^{\circ\:}$) was calculated using the following equation^66^:

17 $$\Delta \:G_{ads}^{^\circ \:} = - RT\:\ln \left( {55.5{K_{ads}}} \right)\:$$

The calculated $\:\:{\Delta\:}{G}_{\mathrm{ads}}^{\circ\:}$ values range from − 36.62 to − 37.75 kJ mol⁻¹ (Table 6). The negative values confirm that the adsorption of MPBA molecules on the carbon steel surface is a spontaneous process^67,68^. The magnitude of $\:{\Delta\:}{G}_{\mathrm{ads}}^{\circ\:}$, lying between − 20 and − 40 kJ mol⁻¹, indicates that the adsorption proceeds through a mixed physicochemical mechanism, involving both electrostatic interactions and chemical bonding between the inhibitor molecules and the metal surface^67,69^.

The slight change in $\:{\Delta\:}{G}_{\mathrm{ads}}^{\circ\:}$ with raising temperature reflects variations in the thermodynamic equilibrium of the adsorption process and indicates that adsorption becomes thermodynamically more favorable at higher temperatures. This behavior is consistent with enhanced inhibitor–metal interactions and improved surface coverage at elevated temperatures^52,68^.

It should be noted that $\:{\Delta\:}{G}_{\mathrm{ads}}^{\circ\:}$ should not be regarded as an absolute criterion for distinguishing between physisorption and chemisorption. Recent studies have demonstrated that adsorption commonly involves a combination of physical and chemical interactions; therefore, $\:{\Delta\:}{G}_{\mathrm{ads}}^{\circ\:}$ values mainly provide qualitative insight into adsorption strength, rather than a definitive classification of the adsorption mechanism^50,67^.

**Table 6:** The thermodynamic adsorption parameters *K*_ads_, ∆*G*^o^_ads_, ∆*H*^o^_ads_, and ∆*S*^o^_ads_ for C-steel in 1.0 M HCl, free of and mixed with MPBA compound.

T, ℃	K_ads_ x10^3^, M^− 1^	∆G^o^_ads_, kJ mol^− 1^	∆H^o^_ads_, kJ mol^− 1^	∆S^o^_ads_, J mol^− 1^ K^− 1^
298	39.87	−36.20		32.3
318	19.61	−36.75	26.56	32.0
338	12.28	−37.75		33.1

**Fig. 10:**
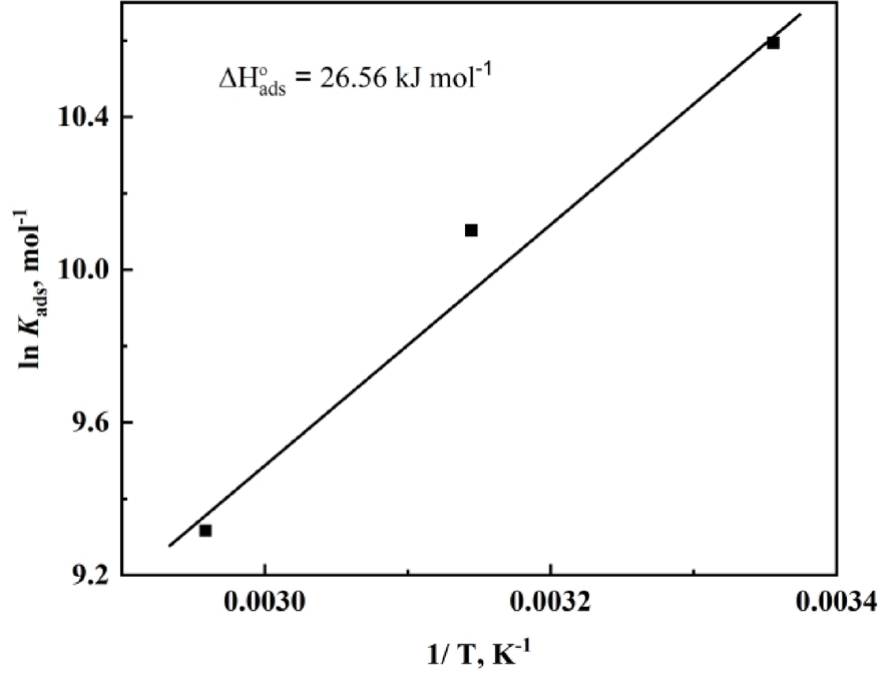
Vant Hoff plots (ln *k* versus 1/T) for the corrosion inhibition of C- steel in 1.0 M HCl.

The Van’t Hoff equation was employed to determine the standard enthalpy of adsorption of MPBA on the carbon steel surface (Δ*H*°_ads_), according to the following relation^66^:

18 $$\ln {K_{ads}} = - \frac{{\Delta \:H_{ads}^{^\circ \:}}}{{RT}} + cons\tan t$$

where $\:{K}_{\mathrm{a}\mathrm{d}\mathrm{s}}$ is the adsorption equilibrium constant, $\:R$ is the universal gas constant, and $\:T$ is the absolute temperature. Figure 10 shows the plot of $\:\mathrm{ln}{K}_{\mathrm{a}\mathrm{d}\mathrm{s}}$ versus $\:1/T$ for the adsorption of MPBA on the carbon steel surface. The slope of the linear fit was used to determine the standard enthalpy of adsorption (Δ*H*°_ads_), which was found to be − 26.56 kJ mol⁻¹ (Table 6). The negative value of Δ*H*°_ads_ indicates that the adsorption of MPBA molecules on carbon steel is an exothermic process^70^. This result accounts for the observed decrease in inhibition efficiency ($\:\eta\:\mathrm{\%}$) with increasing temperature, which can be attributed to the partial desorption of MPBA molecules from the steel surface at elevated temperatures. Furthermore, Δ*H*°_ads_ values in the range of − 20 to − 40 kJ mol⁻¹ are typically associated with physisorption, governed mainly by electrostatic interactions. Since the calculated Δ*H*°_ads_ value is less negative than − 40 kJ mol⁻¹, the adsorption of MPBA on the carbon steel surface is predominantly physical in nature, whereas values exceeding − 40 kJ mol⁻¹ are generally indicative of chemisorption.

However, the entropy of adsorption, Δ*S*^o^_ads_ value, can be deduced from the equation^66^:

19 $$\Delta {G^ \circ }_{ads} = \Delta {H^ \circ }_{ads} - T\Delta {S^ \circ }_{ads}$$

The calculated ΔS°ₐ_d_ₛ values are positive and are between 32.3 and 33.1 J mol⁻¹ K⁻¹ (Table 6), indicating that the adsorption of MPBA molecules is accompanied by a significant increase in disorder at the metal–solution interface during the adsorption process.

### Influence of temperature

**Fig. 11:**
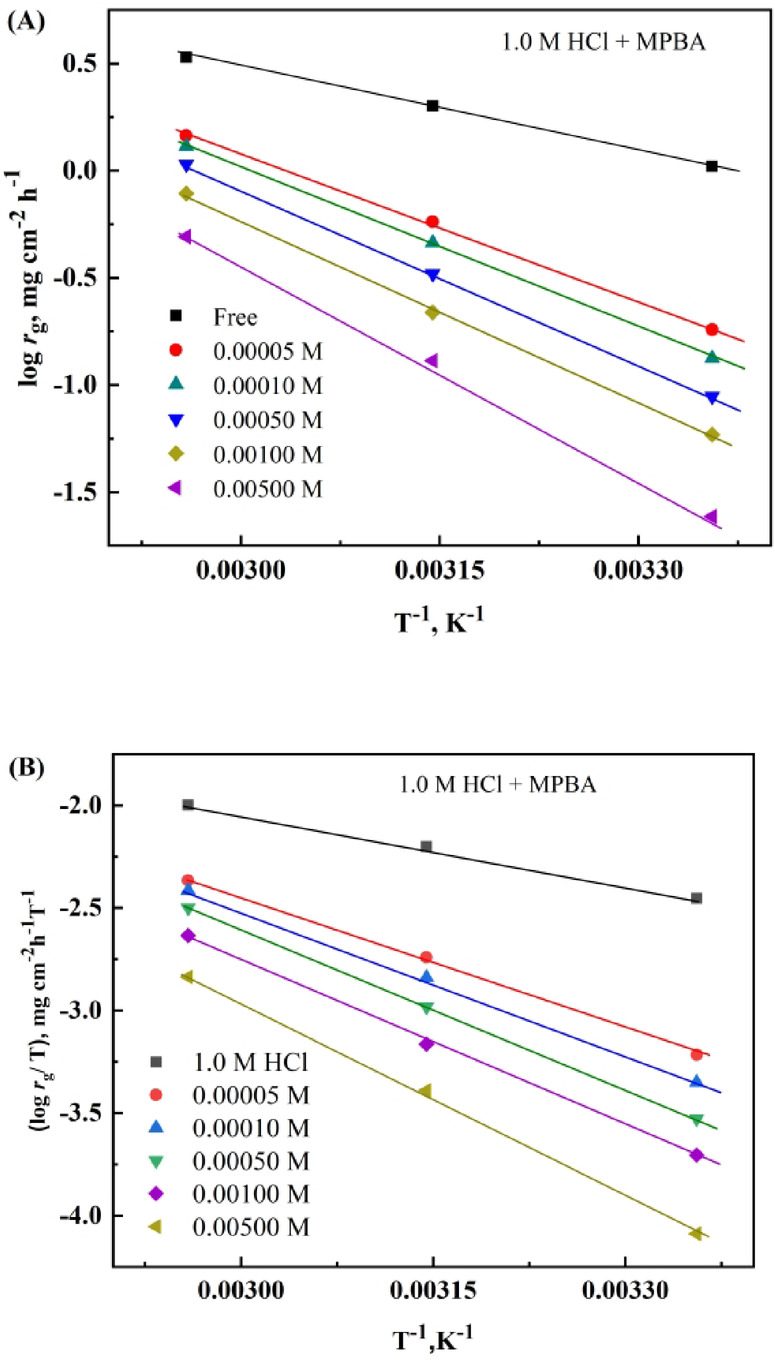
(**A**) Arrhenius and (**B**) transition state plots for the MPBA compound on C-steel in 1.0 M HCl, respectively.

The activation energy (*E*ₐ) for carbon steel corrosion was evaluated using the Arrhenius equation (Eq. 19). The corrosion rates ($\:{r}_{g}$) in dilute HCl solution, in the absence and presence of MPBA, were determined at different temperatures ($\:T$) using the *ML* method. The dependence of $\:{r}_{g}$ on temperature confirms the applicability of the Arrhenius relationship^66^:

20 $$\log {r_g} = - \frac{{{E_a}}}{{2.303RT}} + cons\tan t$$

where $\:{E}_{a}$ is the activation energy and $\:R$ is the universal gas constant. Figure 11A illustrates the plots of $\:\mathrm{log}{r}_{g}$ versus $\:1/T\:$ for both uninhibited and inhibited solutions. Straight-line relationships with correlation coefficients approaching unity were obtained, confirming the validity of the Arrhenius model. The calculated $\:{E}_{a}$ values are summarized in Table 7. The increase in activation energy in the presence of MPBA, together with the observed decrease in inhibition efficiency ($\:\eta\:$) at elevated temperatures, suggests that the adsorption of MPBA molecules on the carbon steel surface is predominantly physical in nature, as higher temperatures tend to weaken physically adsorbed inhibitor layers^71–74^.

The enthalpy of activation (ΔH*) and entropy of activation (ΔS*) for carbon steel corrosion in HCl solution, in the absence and presence of MPBA, were evaluated to describe the energetic and entropic changes associated with the formation of the activated complex during the corrosion process. These kinetic parameters were determined using the transition-state (Eyring) equation^50^:

21 $$\log \left( {\frac{{{r_g}}}{T}} \right) = \log \left( {\frac{R}{{{N_A}h}}} \right) + \frac{{\Delta \:{S^*}}}{{2.303R}} - \frac{{\Delta \:{H^*}}}{{2.303RT}}$$

where $\:{r}_{g}$ is the corrosion rate, $\:T$ is the absolute temperature, $\:R$ is the gas constant, $\:h\:$ is Planck’s constant, and $\:{N}_{A}$ is Avogadro’s number.

Figure 11B presents the plots of $\:\mathrm{l}\mathrm{o}\mathrm{g}({r}_{g}/T)$ versus $\:1/T$ for uninhibited and inhibited acidic solutions. Straight-line relationships were obtained, confirming the applicability of the transition-state model. The slopes of these plots correspond to $\:-{\Delta\:}{\mathrm{H}}^{\mathrm{*}}/\left(2.303R\right)$, and the calculated ΔH* values are summarized in Table 7. The positive ΔH* values indicate that the corrosion process is endothermic, particularly in the presence of the MPBA inhibitor, reflecting the energy required to form the activated complex^51,75^.

The calculated ΔS* value for carbon steel in uninhibited dilute HCl solution was − 171 J mol⁻¹K⁻¹, whereas values ranging from − 122 to − 73 J mol⁻¹K⁻¹ were obtained upon the addition of different MPBA concentrations (Table 7). The negative ΔS* values suggest a decrease in disorder during the formation of the activated complex, which may be attributed to the development of an ordered and stable adsorbed inhibitor layer on the carbon steel surface^50^. Moreover, the progressive increase (less negative values) of ΔS* with increasing MPBA concentration indicates a reduction in the degree of ordering at the activated state, implying that the rate-determining step becomes relatively more associative in nature in the presence of the inhibitor^76–84^.

**Table 7:** The activation thermodynamic functions for C-steel in dilute hydrochloric acid solutions, free of and mixed with various amounts of the MPBA compound.

Conc. of MPBA (M)	E_a_ (kJ mol^− 1)^	∆H*_ads_ (kJ mol^− 1^)	∆S*_ads_ (J mol^− 1^ K^− 1^)
Free	24.56	21.92	−171
0.05	43.71	41.08	−122
0.10	47.71	45.09	−110
0.50	52.23	49.59	−99
1.00	54.19	51.56	−96
5.00	63.05	60.45	−73

### Surface study

**Fig. 12:**
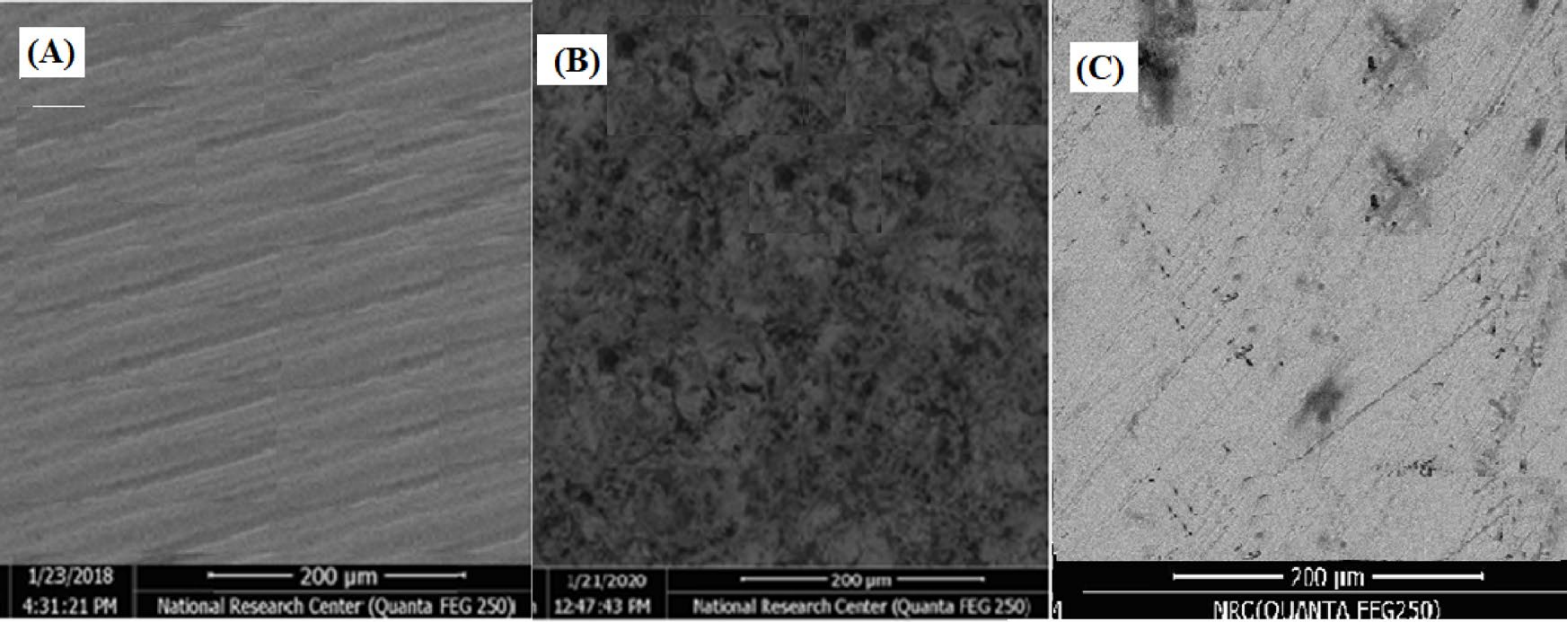
SEM micrographs of C-steel before (**A**), and after (**B**) inundation in free and mixed with MPBA compound in 1.0 M HCl (**C**), successively.

**Fig. 13:**
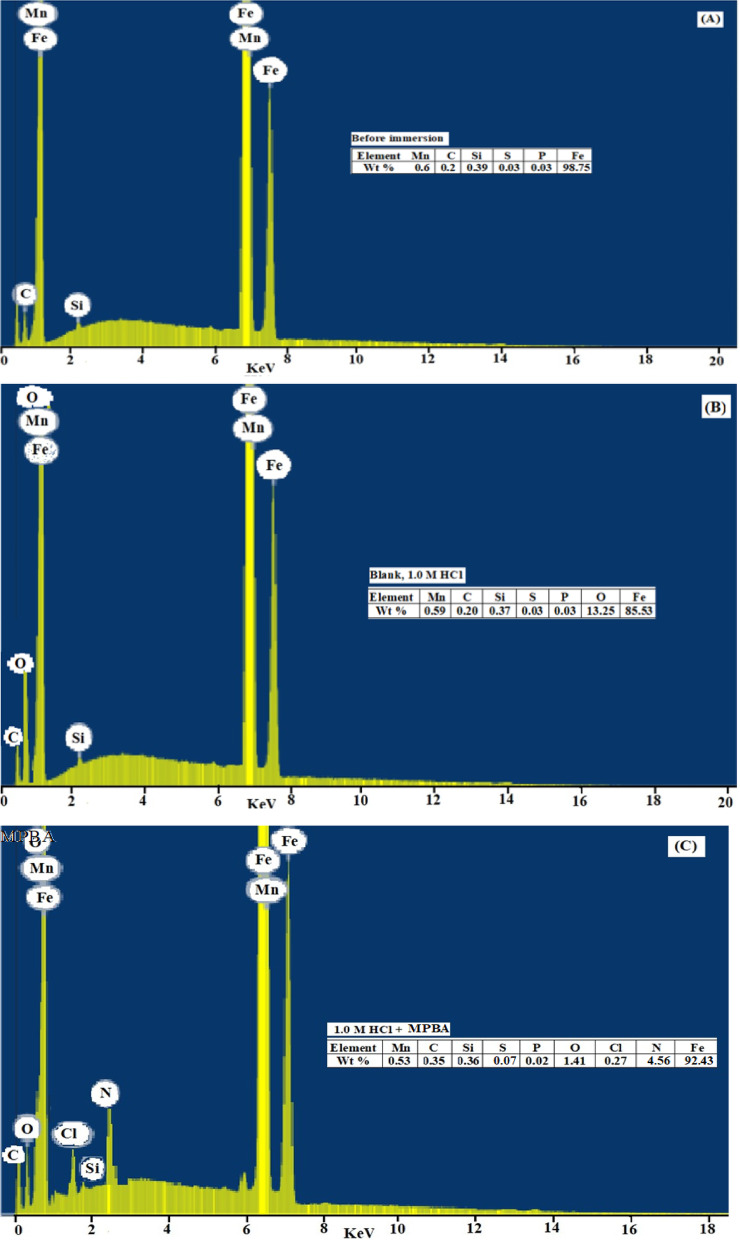
EDS spectra of C-steel before (**A**), and after (**B**) immersion in 1 M HCl, free of and mixed with 5 mM MPBA compound in 1.0 M HCl (**C**), successively.

The surface morphology of selected carbon steel samples was examined using scanning electron microscopy (SEM), complemented by energy-dispersive X-ray spectroscopy (EDS). The SEM and EDS results are shown in Figs. 12 and 13, respectively. Figure 12A depicts the dry carbon steel surface, which is relatively smooth with minor scratches from mechanical polishing. After 4 h immersion in 1.0 M HCl (Fig. 12B), the surface appears severely damaged and rough, covered with corrosion products, reflecting the aggressive action of the acidic medium. The corresponding EDS spectrum (Fig. 13B) shows a reduced iron content to reach 85.53%, consistent with significant metal dissolution.

In contrast, the carbon steel surface exposed to 1.0 M HCl containing 5 mM MPBA for 4 h (Fig. 12C) is considerably less damaged, with fewer corrosion features. This improvement results from the formation of a protective layer due to MPBA adsorption. The EDS analysis (Fig. 13C) confirms a higher iron content (92.43%) compared with the inhibitor-free solution, supporting the conclusion that the adsorbed MPBA layer effectively reduces iron dissolution and enhances corrosion protection.

## Quantum calculations

### DFT computations

**Fig. 14:**
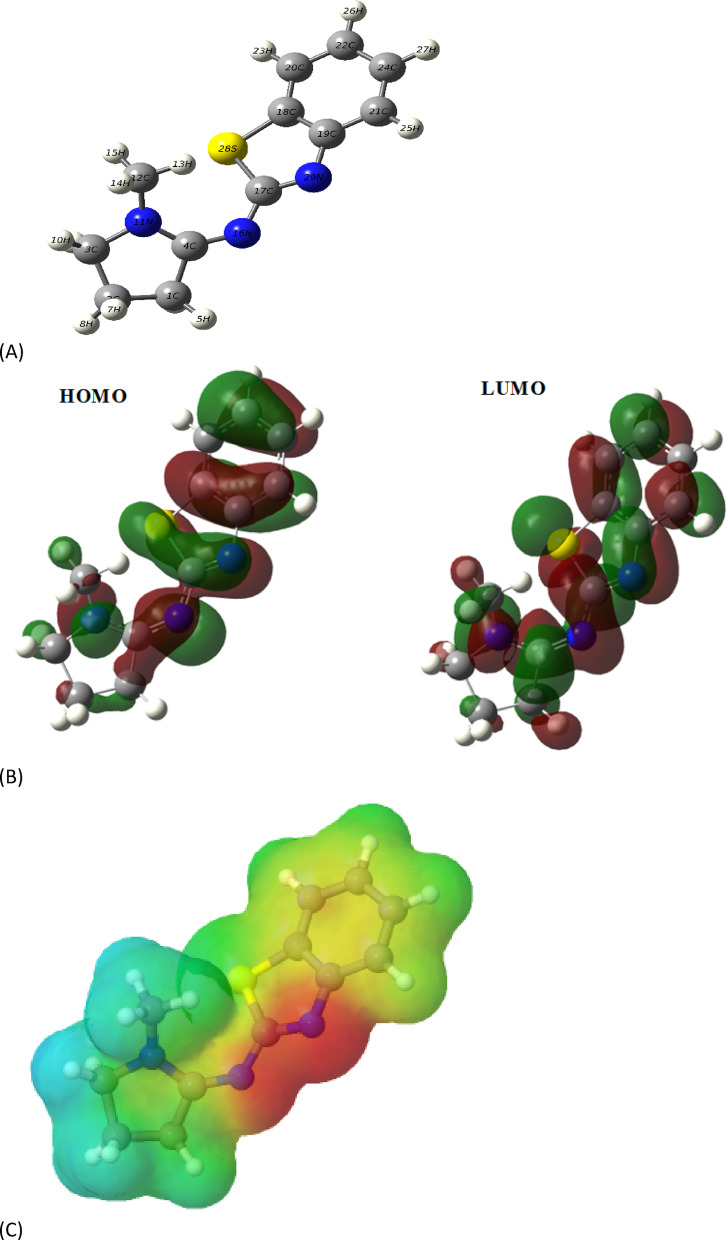
(**A**). The optimized geometry of the MPBA molecule. (**B**). The frontier orbital, FMO, of the MPBA molecule. (**C**). The electrostatic potential map of the MPBA molecule

The MPBA inhibitor was optimized as an aqueous medium using the CPCM model. The optimized geometry (Fig. 14A) provides insights into the molecular structure and properties of the compound. The molecule exhibits C–N and C–C bond lengths of approximately 1.4 Å and 1.5 Å, respectively, consistent with typical single bonds. The C3–N11–C4 bond angle is close to the tetrahedral angle (109.5°), indicating sp³ hybridization of the carbon and nitrogen atoms. The negative dihedral angle (− 24.55°) of the N11–C4–N16–C17 linkage in the 4 s inhibitor suggests partial conjugation within the molecule. In contrast, the dihedral angles of 177.97° and 179.26° in the N11–C4–N16–N17 linkages of the 5 s and 6 s inhibitors indicate an almost planar configuration around the C4–N16 bond.

Frontier molecular orbital (FMO) theory, which involves the Highest Occupied Molecular Orbital (HOMO) and the Lowest Unoccupied Molecular Orbital (LUMO), is widely used to identify the active adsorption sites of inhibitor molecules during their interaction with metal surfaces. In general, corrosion inhibitors act as electron donors to the metal surface, making the HOMO electron density distribution a critical factor. Conversely, the LUMO represents the electron-accepting regions of the organic molecules during adsorption. Moreover, the energy gap (Δ*E*) between the HOMO and LUMO levels is a key parameter for evaluating the reactivity and inhibition performance of corrosion inhibitors. The three-dimensional plots of the frontier molecular orbitals of the MPBA inhibitor are presented in Fig. 14B and C. As shown in Fig. 14B, both the HOMO and LUMO are distributed over almost the entire molecular structure of the MPBA inhibitor. The HOMO is delocalized throughout the molecule, whereas the LUMO is mainly localized on the phenyl hydrazine moiety. This behavior can be attributed to the conjugated electronic system of the phenyl group, where overlapping p orbitals facilitate electron delocalization, leading to preferential localization of the LUMO in this region.

The molecular electrostatic potential (ESP) analysis, depicted in Fig. 14C, is an effective approach for identifying potential reactive sites for electrophilic and nucleophilic attacks. The ESP is closely related to the polarity, dipole moment, and charge distribution of the molecule. In the ESP maps of the MPBA inhibitor, negative potential regions are represented by yellow and red colors, while positive and neutral potentials are indicated by blue and green colors, respectively. Pronounced negative potential regions are mainly located around the nitrogen and sulfur atoms, reflecting their high electronegativity. In contrast, positive potential regions are predominantly associated with the methyl pyrrolidine group. These findings indicate that the electron-rich sites of the MPBA molecule serve as favorable adsorption centers on the metal surface, thereby enhancing its corrosion inhibition performance.

Understanding the distribution of nucleophilic and electrophilic sites is essential for predicting the inhibition mechanism and adsorption behavior of organic inhibitors on metal surfaces. In general, identifying electron-rich and electron-deficient regions within a molecule provides valuable guidance for the rational design of more efficient corrosion inhibitors. The ESP maps offer a clear visual representation of these regions, enabling informed decisions regarding molecular modification, synthesis, and application. Consequently, such theoretical insights contribute to the development of inhibitors with improved selectivity, efficiency, and suitability for specific corrosive environments.

Theoretical calculations were further employed to evaluate the corrosion inhibition performance of the MPBA inhibitor for carbon steel in HCl solution and to correlate the computational results with the experimentally obtained inhibition efficiency data. The calculated quantum chemical parameters, including electronegativity (*η*), global hardness (*σ*), and related descriptors, are summarized in Table 8. The negative value of the HOMO energy (*E*_HOMO_) reflects the strong electron-donating ability of the inhibitor molecule^85,86^, indicating its tendency to transfer electrons to the acceptor metal surface. Furthermore, a lower HOMO–LUMO energy gap (Δ*E*) is generally associated with higher inhibition efficiency, as it implies a lower energy requirement for electron excitation from the highest occupied orbital^87,88^. Accordingly, the MPBA inhibitor, with a Δ*E* value of 4.57 eV, exhibits a high corrosion protection capability, in good agreement with the experimental findings.

Absolute hardness and softness are key indicators for measuring molecular stability and reactivity. Chemical hardness represents the resistance to the deformation or polarization of an atom’s or molecule’s electron cloud during a chemical reaction. A hard molecule exhibits a large energy gap, while a soft molecule shows a smaller energy gap. To evaluate the stability, reactivity, and efficacy of an inhibitor, one must consider hardness (*ɳ*) and softness (*σ*). Organic molecules with low hardness and high softness tend to provide better anti-corrosion properties than those with high hardness and low softness.

**Table 8:** Quantum chemical parameters of the MPBA inhibitor for carbon steel in hydrochloric acid solution.

E_HOMO_, eV	E_LUMO_, eV	ΔE, eV	$\:\sigma\:$ (eV^− 1^)	ɳ (eV)	ΔN (e)
−5.6	−1.03	4.57	0.438	2.285	0.33

Absolute hardness and softness are important descriptors for assessing molecular stability and chemical reactivity. Chemical hardness reflects the resistance of an atom or molecule to deformation or polarization of its electron cloud during a chemical interaction. In general, hard molecules are characterized by a large HOMO–LUMO energy gap, whereas soft molecules possess a smaller energy gap. Therefore, the evaluation of corrosion inhibition performance requires consideration of both absolute hardness (*η*) and softness (σ). Organic inhibitors with lower hardness and higher softness typically exhibit enhanced corrosion inhibition efficiency, as they are more reactive and can more readily interact with the metal surface. Lewis-based molecules, which possess the ability to donate and accept electrons, are particularly effective in corrosion inhibition. Softer molecules generally exhibit superior inhibition performance compared to harder ones, owing to their higher reactivity and stronger tendency to form stable interactions with metal surfaces. This enhanced interaction facilitates the formation of protective adsorbed layers, thereby improving corrosion resistance^89^. In the present study, the MPBA inhibitor, characterized by lower chemical hardness and higher softness, demonstrates favorable corrosion inhibition behavior.

The fraction of electron transfer (Δ*N*) is an important parameter for describing the direction and magnitude of electron exchange between the inhibitor molecule and the metal surface. A positive ΔN value indicates electron transfer from the inhibitor to the metal, whereas a negative value corresponds to electron transfer from the metal to the inhibitor^90^. As summarized in Table 8, the calculated ΔN values obtained using the B3LYP/6-31G(d, p) level of theory are positive, suggesting that electron donation from the MPBA molecule to the metal surface is energetically favorable.

To further elucidate the interaction between the inhibitor molecule and specific active sites on the metal surface, local reactivity descriptors were analyzed. The Fukui function provides valuable information on the susceptibility of different atomic sites to electrophilic and nucleophilic attacks by describing changes in electron density upon electron gain or loss. Accordingly, the Fukui indices $\:{f}^{+}$ and $\:{f}^{-}$ correspond to nucleophilic and electrophilic attacks, respectively. The calculated Fukui function values for the MPBA inhibitor are summarized in Table 9 and were determined using the standard finite-difference approach, allowing the identification of the most reactive atomic centers involved in the adsorption process.

To clarify how a molecule interacts with specific sites on a metal surface, it is essential that the inhibitor preferentially targets the active corrosion sites. The Fukui function provides insight into the local reactivity of a molecule by describing changes in electron density associated with electron gain or loss. Accordingly, the Fukui function is divided into two indices: $\:{f}^{+}$, which characterizes susceptibility toward nucleophilic attack, and $\:{f}^{-}$, which corresponds to electrophilic attack (Table 9). These indices can be calculated using the following relations:

22 $$\:{f}^{+}=\:q\left(N\right)-q\:\left(N+1\right)\:$$

23 $$\:{f}^{-}=\:q\left(N-1\right)-q\:\left(N\right)\:$$

Here, $\:q\left(N\right)$, $\:q(N+1)$, and $\:q(N-1)$ represent the atomic charges of the neutral, anionic, and cationic systems, respectively. A higher $\:{f}^{+}$ value for a given atom indicates a greater susceptibility toward nucleophilic attack, implying that the atom is more prone to electron donation. Conversely, higher $\:{f}^{-}$ values signify a preference for electrophilic attack, suggesting an increased tendency to accept electrons. This distinction provides insight into the interaction mechanism between the inhibitor molecule and the metal surface and plays a crucial role in determining inhibition efficiency.

**Table 9:** Fukui indices for MPBA compound.

Atom	$\:{f}^{+}$	$\:{f}^{-}$	Atom	$\:{f}^{+}$	$\:{f}^{-}$
C (1)	−0.011	−0.002	N (16)	0.045	0.113
C (2)	−0.001	0	C (17)	0.074	0.021
C (3)	−0.005	−0.003	C (18)	0.014	0.044
C (4)	0.121	0.029	C (19)	0.025	0.046
H (5)	0.04	0.021	C (20)	0.031	0.025
H (6)	0.038	0.02	C (21)	0.052	0.046
H (7)	0.017	0.012	C (22)	0.057	0.074
H (8)	0.019	0.014	H (23)	0.03	0.034
H (9)	0.03	0.024	C (24)	0.012	0.036
H (10)	0.023	0.018	H (25)	0.034	0.04
N (11)	0.03	0.037	H (26)	0.033	0.046
C (12)	−0.014	−0.011	H (27)	0.025	0.038
H (13)	0.015	0.012	S (28)	0.129	0.124
H (14)	0.033	0.022	N (29)	0.075	0.093
H (15)	0.028	0.025			

**Table 10:** Optimized parameters for MPBA compound.

Parameter	Value	Parameter	Value
Bond distance, Å	1.46 (C12-N11) 1.35 (N11-C4) 1.52 (C4-C1) 1.48 (N11-C3) 1.31 (C4-N16) 1.83 (C17-S28) 1.31 (C17-29 N) 1.76 (S28-C18) 1.38 (N29-C19) 1.40(C19-C21)	Angle (°)	111.80 (C3-N11-C4) 88.66 (C18-S28-C17) 112.20 (C17-N29-C19) 129.14 (C4-N16-C17)
		Dihedral angle (°)	−24.55 (N11-C4-N16-C17) 175.34 (N16-C17-N29-C19)

As shown in Table 10, the MPBA inhibitor exhibits C (4) and S (28) as the most favorable sites for nucleophilic attack, while N (16) and S (28) are the preferred sites for electrophilic attack, underscoring their key role in the adsorption process.

### Monte Carlo (MC) and Molecular dynamics (MD) simulations

**Fig. 15:**
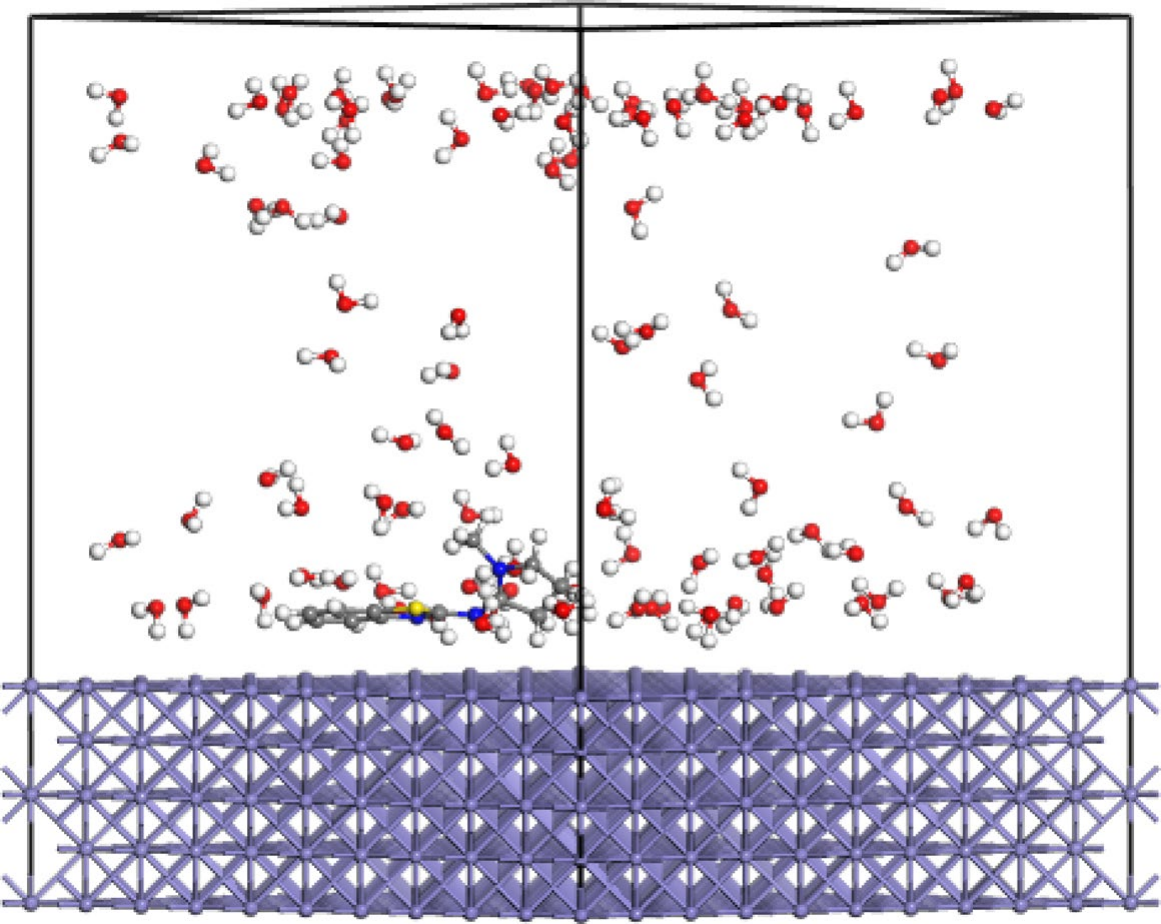
Adsorption of the MPBA inhibitor on the Fe (110) surface.

The effectiveness of the MPBA inhibitor in mitigating corrosion through adsorption on the Fe (110) surface was evaluated using Monte Carlo (MC) simulations. This approach provides detailed energetic information for a system comprising the Fe (110) surface, the inhibitor molecule, and 100 water molecules. The equilibrium adsorption configuration, illustrated in Fig. 15, shows that the MPBA molecule adsorbs preferentially in a nearly parallel orientation relative to the Fe surface, which maximizes surface coverage and intermolecular interactions. The calculated adsorption energy value listed in Table 11 reveals that the MPBA inhibitor exhibits a highly negative adsorption energy (− 463.61 kcal·mol⁻¹), indicating a strong and spontaneous interaction with the Fe (110) surface. Such a markedly negative adsorption energy reflects the high affinity of MPBA toward the iron surface, supporting the formation of a stable and adherent protective film, which is consistent with the experimentally observed high inhibition efficiency.

**Table 11:** Adsorption energy computed for MPBA compound on the Fe (110) surface.

System	Adsorption energy (kcal/mol)
MPBA/100 H_2_O/Fe (110)	−463.61

To further elucidate the nature of the interaction between MPBA molecules and the iron surface, the radial distribution function (RDF), g(r), was employed (Fig. 15). The RDF analysis provides insight into the bonding characteristics and interaction distances between the inhibitor molecules and surface Fe atoms. Peaks appearing at interatomic distances shorter than 3.5 Å are generally attributed to chemisorption, indicating the formation of strong chemical bonds between MPBA and the iron surface. In contrast, peaks observed at distances greater than 3.5 Å correspond to physisorption, which is associated with weaker electrostatic or van der Waals interactions. The presence of pronounced RDF peaks at short distances confirms that the adsorption of MPBA on the Fe (110) surface involves a significant chemisorption contribution, in good agreement with the calculated adsorption energy and electrochemical findings Fig. .

**Fig. 16:**
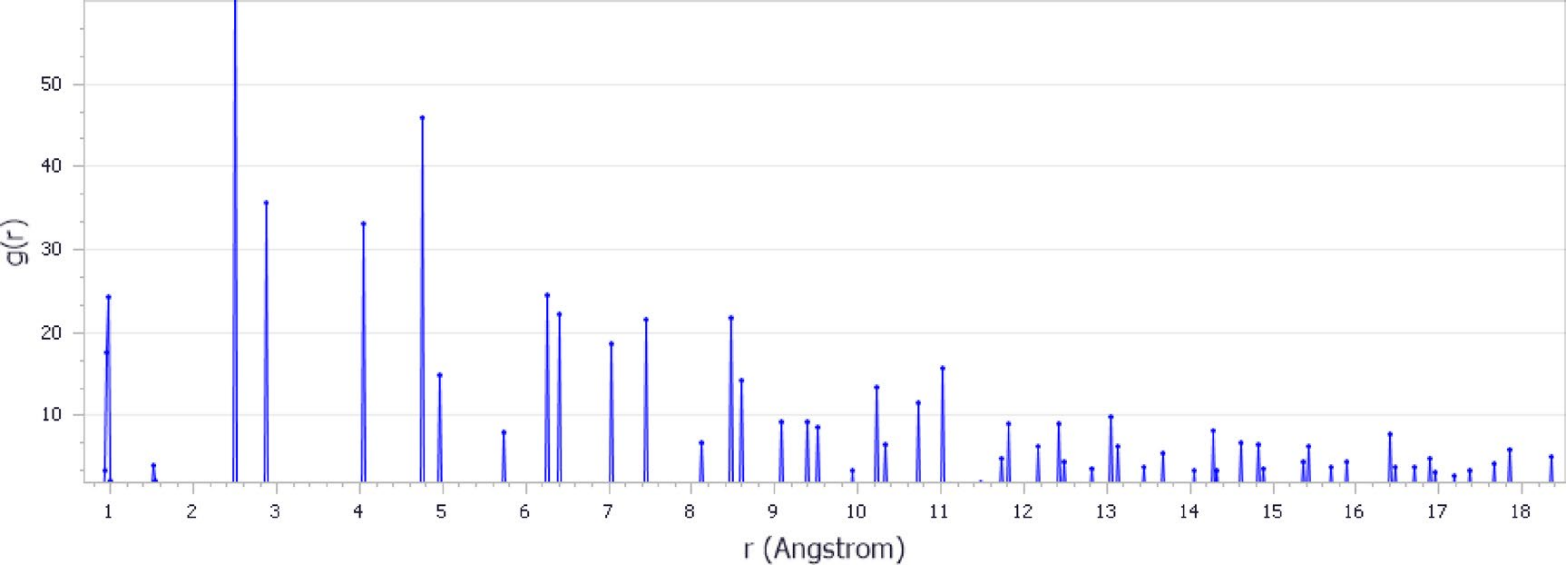
Radial distribution function g(r) analyses for the MPBA inhibitor on the Fe (110) surface.

### Inhibition mechanism

The inhibition behavior of MPBA can be attributed to its adsorption on the carbon steel surface via a combination of physisorption and chemisorption mechanisms. In acidic media, protonation of MPBA facilitates initial electrostatic interaction with the negatively charged metal/solution interface, promoting its approach to the surface. This is followed by donor–acceptor interactions between the lone pair electrons on nitrogen and sulfur atoms, as well as the π-electrons of the benzothiazole ring and imine (C = N) moieties, and the vacant Fe 3 d orbitals, resulting in the formation of a coordinated adsorbed film. The presence of multiple adsorption centers enables quasi-chelating interactions, thereby enhancing adsorption strength and surface coverage. The inhibition efficiency data gained by the different experimental techniques are in agreement with consistent values that prove the good protection process.

Electrochemical impedance spectroscopy (EIS) results support this mechanism, as evidenced by a significant increase in charge transfer resistance (*R*_ct_) and a corresponding decrease in double-layer capacitance (C_dl_). Notably, the inhibition efficiency reaches up to 97.6% at 5 mM, indicating the formation of a compact and highly insulating layer at the metal/electrolyte interface. Furthermore, Tafel polarization curves show a marked decrease in corrosion current density (*i*_corr_), with only slight shifts in corrosion potential (ΔE_corr_ < 85 mV), confirming that MPBA acts as a mixed-type inhibitor affecting both anodic metal dissolution and cathodic hydrogen evolution reactions.

Quantum chemical calculations further support the experimental findings, revealing a highly negative adsorption energy (− 463.61 kcal·mol⁻¹), indicative of a strong and spontaneous interaction between MPBA and the Fe surface. Additionally, the formation of an auto-oxidized species may contribute to a more compact and stable adsorbed layer, thereby enhancing the overall barrier properties of the inhibitor.”

## Conclusions

- The synthesized MPBA compound was successfully characterized using FTIR and ^1^HNMR spectroscopy.
- Experimental investigations demonstrate that MPBA acts as an efficient mixed-type corrosion inhibitor for carbon steel in 2.0 M HCl solution.
- The inhibition efficiency increases with increasing MPBA concentration, while it decreases with rising temperature.
- The corrosion inhibition mechanism is primarily governed by the adsorption of MPBA molecules onto the carbon steel surface, following the Langmuir adsorption isotherm model.
- The observed decrease in surface coverage and inhibition efficiency with increasing temperature suggests that the adsorption process is predominantly physical in nature.
- The relatively high values of the adsorption equilibrium constant (*K*_ads_) and the standard free energy of adsorption (Δ*G*°_ads_) indicate a strong interaction between MPBA molecules and the carbon steel surface.
- The negative values of Δ*G*°_ads_ confirm the spontaneous nature of the adsorption process.
- The moderate magnitudes of ΔG°_ads_ (− 36.62 to − 37.75 kJ mol⁻¹) and Δ*H*°_ads_ (− 8.51 kJ mol⁻¹) suggest that the adsorption mechanism involves a combination of physisorption and chemisorption.
- The adsorption behavior predicted by quantum chemical calculations shows good agreement with the experimental results.

## Supplementary Information

Below is the link to the electronic supplementary material.

Supplementary Information 1.

## Data Availability

The data sets used or analyzed during the current study are included in the text.
