# Supplementary figures and images for: A simple Schiff base compound as mitigator for the destruction of C-steel in HCl aqueous solutions: practical and theoretical studies
Source: Sci Rep. 2026 Jun 20;16:19186. doi: 10.1038/s41598-026-53317-6 (PMC13283215; doi:10.1038/s41598-026-53317-6)

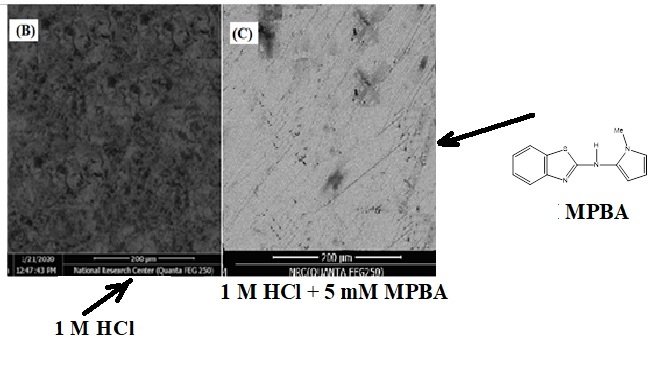

Supplement: Supplementary file 1 — Supplementary Information 1. [file 41598_2026_53317_MOESM1_ESM.jpg]
